# New 1,2,3-Triazole and Dipyridothiazine Hybrids—Synthesis, Analysis, Cytotoxicity and Molecular Docking

**DOI:** 10.3390/biom16030349

**Published:** 2026-02-26

**Authors:** Emilia Martula, Weronika Bagrowska, Paulina Strzyga-Łach, Marta Struga, Małgorzata Latocha, Dariusz Kuśmierz, Małgorzata Jeleń, Beata Morak-Młodawska

**Affiliations:** 1 Department of Organic Chemistry, Faculty of Pharmaceutical Sciences, The Medical University of Silesia, Jagiellońska 4, 41-200 Sosnowiec, Poland; emilia.martula2903@gmail.com (E.M.); manowak@sum.edu.pl (M.J.); 2 Tunneling Group, Biotechnology Centre, Silesian University of Technology, Krzywoustego 8, 44-100 Gliwice, Poland; weronika.bagrowska@polsl.pl; 3 Department of Biochemistry, Medical University of Warsaw, 02-097 Warsaw, Poland; paulina.strzyga-lach@wum.edu.pl (P.S.-Ł.); marta.struga@wum.edu.pl (M.S.); 4 Department of Cell Biology, Faculty of Pharmaceutical Sciences, The Medical University of Silesia, Jedności 8, 41-200 Sosnowiec, Poland; mlatocha@sum.edu.pl (M.L.); dkusmierz@sum.edu.pl (D.K.)

**Keywords:** diazaphenothiazine, cytotoxicity, gene expression analysis, HDAC6, anticancer action

## Abstract

Epigenetic and stress-response pathways play central roles in cancer progression and represent attractive therapeutic targets. In this study, a series of dipyridothiazine–1,2,3-triazole hybrids bearing *p*-fluorophenyl and *p*-trifluoromethylphenyl substituents was synthesized via efficient dipolar cycloaddition reactions. Structural characterization was performed using ^1^H, ^13^C, and ^19^F NMR spectroscopy and high-resolution mass spectrometry. Anticancer activity was evaluated using WST-1 and MTT assays against human cancer cell lines SNB-19 (glioblastoma), C32 (amelanotic melanoma), A549 (lung carcinoma), and MDA-MB-231 and MCF-7 (breast cancer), as well as normal HFF-1 fibroblasts and HaCaT keratinocytes, with doxorubicin and cisplatin as reference drugs. The hybrids **TDT2b** and **TDT3b** containing a *p*-trifluoromethylphenyl moiety showed the highest cytotoxicity and cancer cell selectivity. RT-qPCR analysis of *H3*, *TP53*, *CDKN1A*, *BCL-2*, and *BAX* expression for the lead compound **TDT2b** revealed modulation of chromatin organization, p53-dependent stress responses, apoptosis, and cell cycle regulation. Molecular docking studies with human histone deacetylase 6 (HDAC6) demonstrated favorable binding of **TDT2b** and **TDT3b**, supporting their role as potential epigenetic anticancer agents.

## 1. Introduction

**Figure 1:**
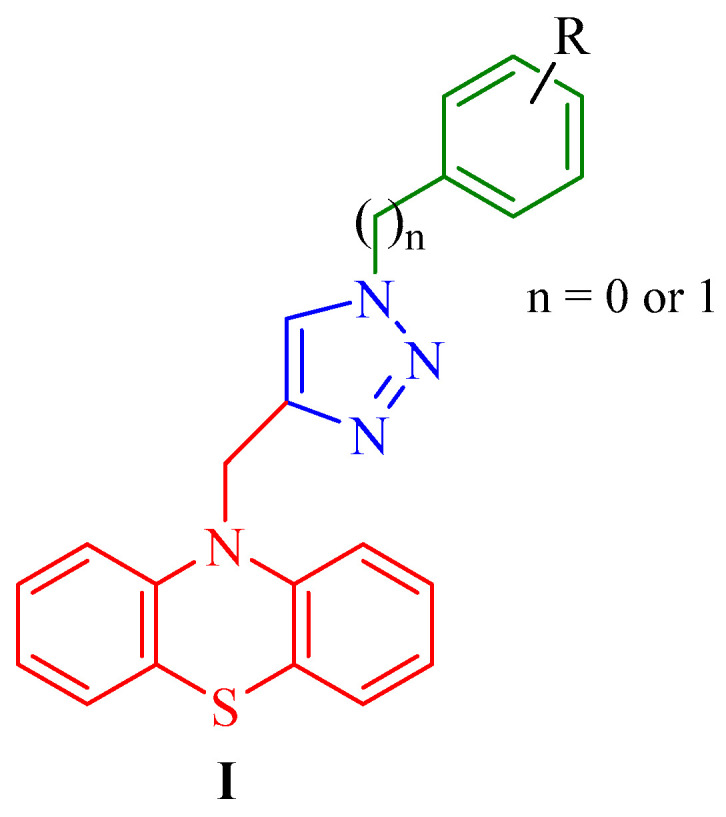
Phenothiazine and 1,2,3-triazole derivatives with documented cytotoxic activities.

Cancer represents one of the most significant challenges in modern medicine and public health, imposing substantial health, social, and economic burdens worldwide [1,2]. The incidence of many cancer types has continued to increase over recent decades, and despite notable advances in diagnostics and therapeutic interventions, cancer remains associated with high mortality rates. Consequently, contemporary oncological research increasingly emphasizes the identification of novel bioactive molecules with improved selectivity and safety profiles, as well as the elucidation of their molecular mechanisms of action. In parallel, the development of advanced diagnostic strategies based on molecular and biomolecular insights has become a central focus of cancer research [3,4]. Natural and synthetic bioactive compounds, primarily heterocyclic systems, play an important role in the development of new scaffolds in medicinal chemistry [5,6]. The 1,2,3-triazole ring represents a highly versatile heterocyclic scaffold due to its nitrogen-rich structure, which enables diverse intermolecular interactions such as hydrogen bonding and coordination with biological targets [7]. Compounds containing this moiety have been shown to modulate multiple biological pathways, resulting in a broad spectrum of pharmacological activities, including antibacterial [8], antioxidant [9], antitubercular [10], antidepressant [7], antimalarial [7], antiviral [7], and most notably anticancer effects [11,12,13,14]. The ability of the 1,2,3-triazole framework to engage key biomolecular targets has driven sustained research interest, as reflected by the increasing number of studies reported annually [15,16,17,18]. In parallel with the extensive interest in 1,2,3-triazole-based scaffolds, several clinically established drugs, including cefatrizine and tazobactam, incorporate this heterocycle within their molecular architectures and are widely applied as antibiotics in clinical practice [19]. Beyond triazoles, other heterocyclic systems have also emerged as privileged structures in medicinal chemistry. Notably, the six-membered 1,4-thiazine ring, featuring nitrogen and sulfur atoms at the 1- and 4-positions, respectively, constitutes the core framework of phenothiazines [20]. These compounds have long been recognized for their neuroleptic, antimanic, and sedative effects in psychiatric therapy. More recently, phenothiazine derivatives have gained renewed attention as versatile platforms for structural optimization, owing to their ability to interact with diverse biological targets and modulate multiple cellular pathways. This polypharmacological profile has positioned 1,4-thiazine-containing compounds as attractive scaffolds for drug repurposing and the development of novel therapeutics, particularly in oncology [21,22,23]. Within this context, dipyridothiazines have emerged as structurally refined analogues of phenothiazines, in which strategic incorporation of pyridine rings enables fine-tuning of electronic properties, lipophilicity, and target selectivity. These modifications may enhance biological activity while mitigating adverse effects commonly associated with classical phenothiazines, thereby underscoring dipyridothiazines as promising next-generation candidates for anticancer drug discovery [24,25]. Recently, there have been reports in the literature about the promising anticancer activity of phenothiazines and 1,2,3-triazoles conjugates [26,27,28] (Figure 1).

These conjugates (I) are built around a phenothiazine core covalently linked to a 1,2,3-triazole ring bearing either benzyl (n = 1) or phenyl (n = 0) substituents, providing a modular platform for structure–activity relationship (SAR) investigations. Systematic variation of substituents (R) on the aromatic rings, including both electron-donating and electron-withdrawing groups, enabled fine-tuning of the electronic and lipophilic properties of the molecules. Notably, the nature of the triazole-linked substituent and the electronic characteristics of the aromatic rings were found to markedly influence cytotoxic potency and selectivity toward cancer cells. These derivatives demonstrated significant antiproliferative activity against multiple cancer cell lines, including gastric (MKN28, MKN45, MGC-803), breast (MDA-MB-231, MDA-MB-468, MCF-7), prostate (PC-3), and hepatocellular carcinoma (HepG2) models. Mechanistic analyses further revealed that the most active compounds preferentially triggered mitochondrial-mediated apoptosis, suggesting a link between molecular structure, intracellular targeting, and induction of programmed cell death.

**Figure 2:**
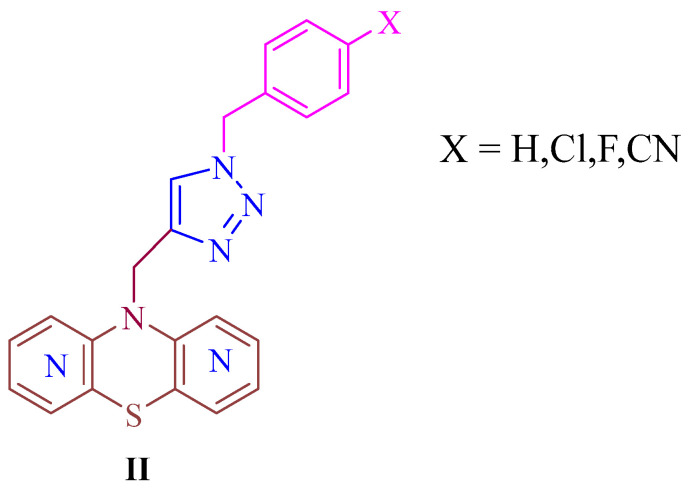
Dipyridothiazine and 1,2,3-triazole hybrids with activity against: glioblastoma SNB-19, colorectal carcinoma Caco-2, lung cancer A549, and breast cancer MDA-MB231.

Consistent SAR trends were also identified for modified dipyridothiazines (**II**), previously reported and illustrated in Figure 2, where conjugation with a five-membered 1,2,3-triazole ring led to a marked enhancement of anticancer activity against glioblastoma (SNB-19), colorectal carcinoma (Caco-2), lung cancer (A549), and breast cancer (MDA-MB-231) cell lines [29]. This group of derivatives included compounds exhibiting pronounced broad-spectrum antiproliferative effects, with IC_50_ values ranging from 0.25 to 4.66 μM, while simultaneously displaying low cytotoxicity toward normal human fibroblasts (NHFF). At the molecular level, gene expression analyses of *H3*, *TP53*, *CDKN1A*, *BCL-2*, and *BAX* revealed that the most active compounds induced mitochondrial-mediated apoptosis, as evidenced by a significant disruption of the *BCL-2/BAX* expression ratio in favor of pro-apoptotic signaling [29].

The present study was motivated by compelling literature reports [26,27,28] as well as our previous experience with azaphenotiazines and azaphenothiazines–1,2,3-triazole hybrid systems bearing benzyl substituents [29,30,31]. Building on these findings, we designed and synthesized a new series of dipyridothiazine–1,2,3-triazole hybrids incorporating a conformationally rigid phenyl substituent functionalized with CF_3_ and F groups at the *para* position of the aromatic ring. This structural modification was intended to probe the influence of increased rigidity and electron-withdrawing substituents on anticancer activity. These derivatives were evaluated for cytotoxic activity against a panel of human cancer cell lines, including glioblastoma (SNB-19), melanoma (C-32), breast cancers (MCF-7 and MDA-MB-231), and lung cancer (A549), as well as normal human fibroblasts (HFF-1) and keratinocytes (HaCaT). To investigate the underlying molecular mechanisms, gene expression analyses were performed for the most active compound using RT-qPCR. In addition, molecular modeling studies explored interactions with histone deacetylase 6 (HDAC6), a cytoplasmic enzyme that regulates protein acetylation and cellular stress responses, highlighting potential mechanisms contributing to the observed anticancer effects.

## 2. Materials and Methods

### 2.1. Chemical Part

^1^H, ^13^C, and ^19^F NMR spectra were recorded on a Bruker Ascend™ 600 MHz spectrometer (Bruker, Rheinstetten, Germany) using CDCl_3_ as the solvent. High-resolution mass spectra (HRMS) were obtained by electrospray ionization (ESI) on a Bruker Impact II mass spectrometer (Bruker, Billerica, MA, USA). Melting points of solid compounds were determined in open capillaries using a Boetius melting point apparatus (Stuart Equipment, Stone, UK) and are reported without correction.

All reagents and solvents were purchased from commercial suppliers and used as received without further purification. Phenyl azides, propargyl bromide, potassium tert-butoxide, and copper(I) iodide were obtained from Sigma-Aldrich (Burlington, MA, USA). Toluene, N,N-dimethylformamide (DMF), and chloroform were purchased from POCh (Gliwice, Poland).

The parent dipyridothiazines: 10*H*-1,6-diazaphenothiazine (**DT1**), 10*H*-1,8-diazaphenothiazine (**DT2**), 10*H*-2,7-diazaphenothiazine (**DT3**), and 10*H*-3,6-diazaphenothiazine (**DT4**) were synthesized according to previously reported procedures [32,33,34,35] via a Smiles rearrangement carried out in boiling dimethylformamide (DMF) using the corresponding dipyridyl sulfides. The 10-propynyl derivatives (**PDT1**–**PDT4**) were prepared following literature methods [29] through an alkylation reaction with propargyl bromide in the presence of potassium *tert*-butoxide, in DMF at room temperature.

*General procedure for synthesis of 1,2,3-triazole and dipyridothiazine hybrids (TDT1a,b-TDT4a,b)*

In a 25 mL round-bottom flask, anhydrous toluene (10 mL) was combined with the appropriate 10-propargyl dipyridothiazine derivative (0.3 mmol) (**PDT1**–**PDT4**), a catalytic amount of copper(I) iodide, and the selected *p*-fluorophenyl or *p*-trifluoromethylphenyl azide (0.33 mmol). The reaction mixture was placed on a magnetic stirrer and heated at 90 °C for 72 h under continuous stirring. Upon completion of the reaction, the mixture was allowed to cool to room temperature, and the solvent was removed under reduced pressure using a rotary evaporator. The resulting crude product was subsequently purified by column chromatography with aluminum oxide, using chloroform as eluent. The following hybrid structures were obtained using the following methods:

- 10-((1-(4-fluorophenyl)-1*H*-1,2,3-triazol-4-yl)methyl)-10*H*-dipyrido [2,3-b:2′,3-e][1,4]thiazine (**TDT1a**)

(90.3 mg; 80%), mp = 110–112 °C

^1^H NMR (CDCl_3_) δ: 5.37 (s, 2H, CH_2_), 6.99 (dd, *J* = 5.3 Hz, 1H), 7.22 (m, 2H, 2Hph), 7.38 (m, 1H), 7.40 (m, 1H), 7.71 (m, 2H, 2Hph), 7.98 (m, 1H), 8.17 (dd, *J* = 4.9 Hz, 1H), 8.21 (s, 1H, H_TR_), 8,22 (s, 1H). ^13^C NMR (CDCl_3_) δ: 41.92 (CH_2_), 112.79, 116.81 (d, 2*J_C-F_* = 19.5 Hz, Co), 118.70, 122.78 (d, 3*J_C-F_* = 9 Hz, Cm), 123.33, 124.22, 126.0, 133.04, 133.94, 136.05 (d, 4*J_C-F_* = 3 Hz, Cp), 142.09, 143.21, 146.20, 150.46, 152.01, 162.64 (d, 1*J_C-F_* = 248 Hz C-F). ^19^F NMR (CDCl_3_) δ: −110,52. HR MS (EI) *m*/*z* [C_19_H_13_FN_6_S + H], calc. 377.0989; Found: 377.0986.

b. 10-((1-(4-fluorophenyl)-1H-1,2,3-triazol-4-yl)methyl)-10*H*-dipyrido [3,2-b:3′,4′-e][1,4]thiazine (**TDT2a**)

(96 mg; 85%), mp. = 120–122 °C

^1^H NMR (CDCl_3_) δ: 5.35 (s, 2H, CH_2_), 6.91 (dd, *J* = 5.3 Hz, 1H), 7.23 (m, 4H), 7.43 (m, 2H, 2H_ph_), 8.07 (dd, *J* = 4.8 Hz, 1H), 8.17 (s, 1H, H_TR_), 8.37 (broad s, 1H). ^13^C NMR (CDCl_3_) δ: 41.05 (CH_2_), 122.33, 116.76 (d, 2*J_C-F_* = 19.5 Hz, Co), 120.26, 122.77 (d, 3*J_C-F_* = 9 Hz, Cm), 125.22, 126.01, 133.12, 133.14, 134.92 (d, 4*J_C-F_* = 3 Hz, Cp), 142.44, 143.21, 146.72, 150.68, 152.01, 162.52 (d, 1*J_C-F_* = 249 Hz C-F). ^19^F NMR (CDCl_3_) δ: −113.30. HR MS (EI) *m*/*z* [C_19_H_13_FN_6_S + H], calc. 377.0989; Found: 377.0987.

c. 10-((1-(4-fluorophenyl)-1*H*-1,2,3-triazol-4-yl)methyl)-10*H*-dipyrido [3,4-b:3′,4′-e][1,4]thiazine (**TDT3a**)

(101.6 mg; 90%), mp = 115–117 °C

^1^H NMR (CDCl_3_) δ: 5.26 (s, 2H, CH_2_), 6.74 (d, *J* = 5.1 Hz, 1H), 7.02 (d, *J* = 5.1 Hz, 1H), 7.22 (m, 2H, 2H_ph_), 7.68 (m, 2H, 2H_ph_), 7.85 (s, 1H, H_TR_), 8.07 (s, 1H), 8.09 (s, 1H), 8.14 (d, *J* = 5.1 Hz, 1H), 8.21 (d, *J* = 5.1 Hz, 1H). ^13^C NMR (CDCl_3_) δ: 44.36 (CH_2_), 109.57, 116.94 (d, 2*J_C-F_* = 19.5 Hz, Co), 117.17, 120.37, 121.70, 122.65 (d, 3*J_C-F_* = 9 Hz, Cm), 132.36, 132.77 (d, 4*J*_C-F_ = 3 Hz), 135.21, 137.97, 142.72, 144.96, 146.10, 149.28, 150.05, 162.31 (d, 1*J*_C-F_ = 249 Hz C-F). ^19^F NMR (CDCl_3_) δ: −111.08. HR MS (EI) *m*/*z* [C_19_H_13_FN_6_S + H], calc. 377.0989; Found: 377.0982.

d. 10-((1-(4-fluorophenyl)-1*H*-1,2,3-triazol-4-yl)methyl)-10*H*-dipyrido [2,3-b:4′,3′-e][1,4]thiazine (**TDT4a**)

(92.6 mg; 82%), oil.

^1^H NMR (CDCl_3_) δ: 5.18 (s, 2H, CH_2_), 6.72 (m, 1H), 6.98 (m, 1H), 7.08 (m, 1H), 7.21 (m, 4H, 2H_ph_), 7.72 (m, 2H, 2H_ph_), 7.92 (s, 1H, H_TR_), 8.05 (m, 1H). ^13^C NMR (CDCl_3_) δ: 41.83 (CH_2_), 114.83.57, 115.73 (d, 2*J_C-F_* = 19.5 Hz, C*o*), 116.12, 118.59, 123.22, 129.08 (d, 3*J_C-F_* = 10 Hz, Cm), 130.00, 131.77 (d, 4*J*_C-F_ = 3 Hz), 134.36, 135.79, 142.72, 143.89, 144.89, 145.49, 153.00, 162.77 (d, 1*J*_C-F_ = 249 Hz C-F). ^19^F NMR (CDCl3) δ: −110.49. HR MS (EI) *m*/*z* [C_19_H_13_FN_6_S + H], calc. 377.0989; Found: 377.0985.

e. 10-((1-(4-(trifluoromethyl)phenyl)-1*H*-1,2,3-triazol-4-yl)methyl)-10*H*-dipyrido [2,3-b:2′,3-e][1,4]thiazine (**TDT1b**)

(103,6 mg; 81%), mp = 180–183 °C

^1^H NMR (CDCl_3_) δ: 5.31 (s, 2H, CH_2_), 6.83 (m, 1H), 6.96 (m, 1H), 7.19 (m, 1H), 7.46 (d, *J *= 7.8 Hz, 1H), 7.80 (d, *J *= 12 Hz, 2H_ph_), 7.87 (d, *J *= 12 Hz, 2H_ph_), 7.89 (d, *J *= 4.2 Hz, 1H), 8.04 (d, *J *= 4.2 Hz, 1H), 8.17 (s, 1H, H_TR_). ^13^C NMR (CDCl_3_) δ: 41.85 (CH_2_), 116.80, 118.62, 120.55, 121.36, 122.20 (d, *J_C-F_* = 12 Hz), 124.41, 125.31, 127.06 (m, *J_C-F_* = 12 Hz), 128.24, 129.05, 134.61, 138.40, 142.95, 143.96, 144.92, 145.78, 152.04. ^19^F NMR (CDCl_3_) δ: −62.61. HR MS (EI) *m*/*z* [C_20_H_13_F_3_N_6_S + H], calc. 427.0953; Found: 427.0944.

f. 10-((1-(4-(trifluoromethyl)phenyl)-1*H*-1,2,3-triazol-4-yl)methyl)-10*H*-dipyrido [3,2-b:3′,4′-e][1,4]thiazine (**TDT2b**)

(108.7 mg; 85%), mp = 208–210 °C

^1^H NMR (CDCl_3_) δ: 5.44 (s, 2H, CH_2_), 6.85 (m, 1H), 6.94 (m, 1H), 7.26 (m, 1H), 7.79 (d, *J *= 11 Hz, 2H_ph_), 7.88 (d, *J *= 11 Hz, 2H_ph_), 8.06 (m, 2H), 8.07 (s, 1H, H_TR_), 8.33 (s, 1H). ^13^C NMR (CDCl_3_) δ: 41.70 (CH_2_), 114.79, 118.92, 120.56, 121.20, 122.60 (d, *J_C-F_* = 12 Hz), 124.43, 127.00, 127.04 (q, *J_C-F_* = 12 Hz), 130.60, 132.83, 134.52, 134.67, 139.41, 143.18, 145.36, 145.63, 152.69. ^19^F NMR (CDCl_3_) δ: −62.68. HR MS (EI) *m*/*z* [C_20_H_13_F_3_N_6_S + H], calc. 427.0953; Found: 427.0990.

g. 10-((1-(4-(trifluoromethyl)phenyl)-1*H*-1,2,3-triazol-4-yl)methyl)-10*H*-dipyrido [3,4-b:3′,4′-e][1,4]thiazine (**TDT3b**)

(119 mg; 93%), mp = 170–172 °C

^1^H NMR (CDCl_3_) δ: 5.27 (s, 2H, CH_2_), 6.72 (m, 1H), 7.04 (m, 1H), 7.80 (d, *J* = 9 Hz, 2H_ph_), 7.89 (d, *J* = 9 Hz, 2H_ph_), 7.97 (s, 1H, H_TR_), 8.11 - 8.21 (m, 4H). ^13^C NMR (CDCl_3_) δ: 44.30 (CH_2_), 109.60, 118.90, 120.53 (q, *J*_C-F_ = 12 Hz), 121.53, 122.52, 127 (q, *J*_C-F_ = 12 Hz), 131.23 (q, *J*_C-F_ = 12 Hz), 133.62, 135.25, 139.00, 143.63, 145.03, 146.20, 149.34, 149.95, 162.53. ^19^F NMR (CDCl_3_) δ: −62.63. HR MS (EI) *m*/*z* [C_20_H_13_F_3_N_6_S + H], calc. 427.0953; Found: 427.0956.

h. 10-((1-(4-(trifluoromethyl)phenyl)-1*H*-1,2,3-triazol-4-yl)methyl)-10*H*-dipyrido [2,3-b:4′,3′-e][1,4]thiazine (**TDT4b**)

(115.3 mg; 90%), oil

^1^H NMR (CDCl_3_) δ: 5.21 (s, 2H, CH_2_), 6.72 (m, 1H), 7.01 (m, 1H), 7.07 (m, 1H), 7.82 (d, *J* = 8.4 Hz, 2H, H_ph_), 7.92 (d, *J* = 8.4 Hz, 2H, H_ph_), 8.06 (s, 1H, H_TR_), 8.07 (m, 1H), 8.13 (m, 1H), 8.22 (m, 1H). ^13^C NMR (CDCl_3_) δ: 44.43 (CH_2_), 109.13, 118.90, 120.36 (q, *J*_C-F_ = 16 Hz), 121.24, 121.46, 122.33, 127.25 (q, *J*_C-F_ = 3 Hz), 131.30 (q, *J*_C-F_ = 33 Hz), 137.41, 138.96, 143.56, 144.41, 145.24, 148.02, 149.59, 154.60, 161.04. ^19^F NMR (CDCl_3_) δ: −62.69. HR MS (EI) *m*/*z* [C_20_H_13_F_3_N_6_S + H], calc. 427.0953; Found: 427.0944.

Spectral data of the described compounds are included in the Supplementary Materials.

### 2.2. Biological Evaluation

All newly synthesized hybrids (**TDT1a,b**–**TDT4a,b**) were evaluated for their cytotoxic activity using a panel of cultured cell lines, including: SNB-19 (human glioblastoma; DSMZ—German Collection of Microorganisms and Cell Cultures, Braunschweig, Germany), C32 (human amelanotic melanoma; ATCC, Manassas, VA, USA), A549 (human lung carcinoma; ATCC, Manassas, VA, USA), MDA-MB-231 and MCF-7 (human breast cancer cell lines; ATCC, Manassas, VA, USA), as well as normal human cells HFF-1 (human fibroblasts; ATCC, Manassas, VA, USA) and HaCaT (human keratinocytes; ATCC, Rockville, MD, USA). Cells were cultured at 37 °C in a humidified atmosphere containing 5% CO_2_. For cytotoxicity assays, cells were seeded in 96-well plates at a density of 1 × 10^4^ cells per well in 100 μL of DMEM supplemented with 10% fetal bovine serum (FBS) and penicillin.

Biological activity studies were conducted by two research teams: the Medical University of Silesia and the Medical University of Warsaw, using the WST-1 assay and the MTT assay, respectively.

#### 2.2.1. WST-1 Cell Assay

Cell viability, cytotoxicity, and proliferation in response to the test compounds were evaluated using the WST-1 assay (Roche Diagnostics, Mannheim, Germany) [29]. Cells (SNB-19, C-32, MCF-7, and HFF-1) were exposed to concentrations of 0–100 μg/mL of each compound for 72 h. Further, 10 μL of WST-1 reagent was added and incubated for 1 h. Absorbance was recorded at 450 nm with a reference wavelength of 600 nm using a microplate reader. All results represent the mean values from at least two independent experiments, each conducted in triplicate.

#### 2.2.2. MTT Cell Assay

To assess the cytotoxic activity of the newly synthesized compounds, a preliminary MTT assay (3-(4,5-dimethylthiazol-2-yl)-2,5-diphenyl-2H-tetrazolium bromide) [36] was conducted on two cancer cell lines: A549 and MDA-MB-231, as well as on the normal HaCaT keratinocyte line. Cells were seeded in 96-well plates at 1 × 10^4^ cells per well and allowed to stabilize before treatment with varying concentrations of the test compounds. After 24 h at 37 °C and 5% CO_2_, MTT reagent was added, and the resulting formazan was dissolved in a 1:1 mixture of isopropanol and DMSO. Absorbance was measured at 570 nm (reference 600 nm) using a UVM 340 reader, and IC_50_ values were calculated with GraphPad Prism 8.

#### 2.2.3. The RT-qPCR Method

Gene expression levels of *H3*, *TP53*, *CDKN1A*, *BCL-2*, and *BAX* were quantified using real-time RT-qPCR performed on an OPTICON™ DNA Engine (MJ Research, USA) with the QuantTect^®^ SYBR^®^ Green RT-PCR Kit (Qiagen). Cells were treated with compound **TDT2b** at a concentration of 0.5 μg/mL for 24 h prior to RNA isolation. Total RNA was extracted using the Quick-RNA™ MiniPrep Kit (Zymo Research), and its integrity was confirmed by electrophoresis on a 1.2% agarose gel stained with ethidium bromide. RNA quantity and purity were determined spectrophotometrically using an HP845 instrument (Hewlett Packard). Statistical evaluation was carried out using Statistica 8.0 software, and results are presented as mean ± standard error (SE).

### 2.3. Molecular Docking

#### 2.3.1. Structure Selection and Preparation

The structure of histone deacetylase 6 (HDAC6) was selected for in silico analysis. The structure was downloaded from the Protein Data Bank (PDB ID: 5EDU) [37].

Only the CD2 domain (residues Ser479–Arg835) containing a Zn^2+^ ion in the active site was selected for analysis. The inhibitor located in the active site was manually removed. A water molecule stabilizing the ion in the ligand-free structure was also added to the structure. The position of this water molecule was determined based on an analysis of the apo HDAC6 structure from Danio rerio (PDB ID: 5EEM).

Hydrogen atoms were added to the structure at pH 7.4 using the H++ server [38]. His651 was protonated in the HIE tautomeric form (protonated at NE2 and lacking the HD1 hydrogen) to allow coordination and stabilization of the zinc ion. The structure for molecular dynamics simulations was subsequently prepared using the AMBER MCPB.py [39] and LEaP [40] tools, employing the TIP3P water model and the FF14SB force field. Geometry optimization was performed using Gaussian 09 (revision D.02) [41]. The residues His651, Asp649, Asp742, and one water molecule were designated as zinc-coordinating ligands. The ionic charge of the Zn atom was set to +2.

#### 2.3.2. Molecular Dynamics Simulation

A short molecular dynamics simulation was performed using the AMBER 24 software package [42]. Energy minimization and equilibration were carried out following the protocol described by Daniel R. Roe and Bernard R. Brooks (“A protocol for preparing explicitly solvated systems for stable molecular dynamics simulations”) [43]. The tenth stage, corresponding to the production run, was extended from 1 ns to 10 ns. The system was heated gradually from 0 K to 300 K. Production molecular dynamics simulations were conducted with a 2 fs timestep using Langevin dynamics (collision frequency 5 ps^−1^) at 300 K. Long-range electrostatics were calculated using the Particle Mesh Ewald (PME) method with a 9 Å cutoff. All bonds involving hydrogen atoms were constrained using the SHAKE algorithm. Pressure was maintained at 1 atm using the Monte Carlo barostat. Trajectory coordinates were recorded every 1 ps. The coordinates of the protein from the final frame were saved as a separate PDB file and used for subsequent analysis.

#### 2.3.3. Molecular Docking

Two tested compounds: **TDT2b**, **TDT3** were docked to the last frame obtained in the molecular dynamics simulation. Docking was performed using AutoDock Vina 1.2.3 [44] together with AutoDockTools4 [45]. The center and dimensions of the docking box were set in the vicinity of the active site: size_x = 20 Å, size_y = 20 Å, size_z = 20 Å, centre_x = 42.57 Å, centre_y = 38.25 Å, and centre_z = 45.16 Å. The ligands were prepared using the mk_prepare_ligand.py script, while the receptor was prepared using mk_prepare_receptor.py from the Meeko/AutoDock Suite package. Docking was performed using the Vina scoring function, with the parameter—exhaustiveness = 32 to thoroughly search the conformer space and—num_modes = 10 to generate the ten most favorable positions for each ligand. All output files were saved in pdbqt format, compatible with AutoDock Vina. Protein–ligand interactions were performed using the Protein–Ligand Interaction Profiler (PLIP) server [46].

### 2.4. In Silico Pharmacokinetic Analysis

In silico analyses were performed to predict key physicochemical and pharmacokinetic properties of the tested compounds using the SwissADME web server [47]. Molecular descriptors, including molecular weight, hydrogen bond donors and acceptors, molar refractivity, and number of rotatable bonds, were calculated. Lipophilicity was evaluated using multiple predictive models provided by the platform. In addition, drug-likeness was assessed by examining the compliance of the compounds with established rules, namely Lipinski’s Rule of Five, the Ghose filter, and the Veber criteria, to estimate their oral bioavailability and suitability as potential drug candidates.

## 3. Results and Discussion

### 3.1. Chemical Part

**Scheme 1:**
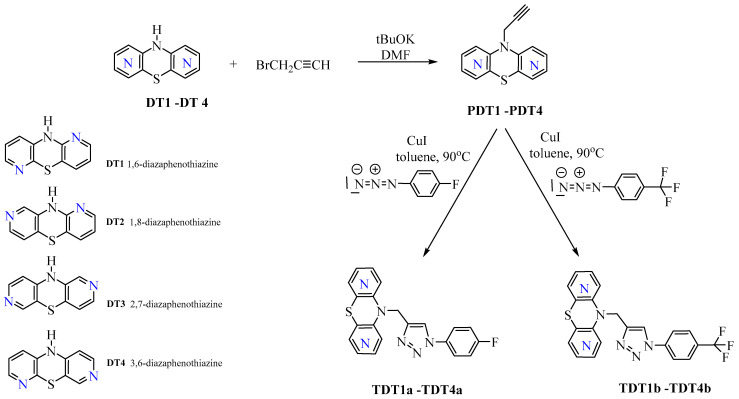
Synthetic routes used for the preparation of the title hybrid compounds.

The synthetic approach employed for the preparation of the novel diazaphenothiazine–1,2,3-triazole hybrids was based on the use of dipyridothiazines **DT1**–**DT4** as key scaffolds for the construction of the 1,2,3-triazole moiety, as outlined in Scheme 1. The parent compounds: 10*H*-1,6-, 1,8-, 2,7-, and 3,6-diazaphenothiazines (**DT1**–**DT4**) were initially converted into the corresponding 10-propynyl derivatives (**PDT1**–**PDT4**) via reaction with 2-propynyl bromide, following previously reported procedures [29,32,33,34,35]. Subsequently, these intermediates were subjected to CuI-catalyzed 1,3-dipolar cycloaddition (“click chemistry”) with selected organic azides (*p*-fluorophenyl azide, *p*-trifluoromethylphenyl azide) in toluene, affording the substituted triazole derivatives (**TDT1a,b**–**TDT4a,b**) with good yields ranging from 80% to 93% (Scheme 1).

The crude reaction mixtures obtained after completion of the synthetic procedures were subjected to purification by column chromatography, which enabled the isolation of the target compounds (**TDT1a,b**–**TDT4a,b**). The chemical structures of the final products were unambiguously elucidated using ^1^H, ^13^C, ^19^F NMR spectroscopy, as well as high-resolution mass spectrometry (HRMS). The spectral data are provided in the Supplementary Materials.

### 3.2. Biological Evaluation

Based on our previous studies showing strong antiproliferative activity of dipyridothiazine derivatives [27,30,31,32,33] and literature reports on the anticancer potential of 1,2,3-triazole-containing compounds [17,26,28], we combined these two scaffolds. This approach was expected to produce synergistic molecular effects. Dipyridothiazines disrupt mitochondrial function, induce apoptosis by altering the BCL-2/BAX balance, and regulate cell cycle and survival pathways [29,34,35]. Triazole moieties improve molecular stability, enhance target binding, and enable selective inhibition of epigenetic regulators, including HDAC6 [23]. We therefore hypothesized that the integration of a 1,2,3-triazole unit into the dipyridothiazine core could simultaneously affect mitochondrial integrity, promote apoptotic signaling, and modulate epigenetic pathways, leading to a multitarget mechanism of action. Based on the above rationale, the designed, synthesized hybrids were evaluated for their in vitro antiproliferative activity on selected human cancer cell lines: SNB-19 (human glioblastoma), C32 (human amelanotic melanoma), A549 (human lung carcinoma), MDA-MB-231 and MCF-7 (human breast cancer cell lines) and normal cells: HFF-1 (human fibroblasts) and HaCaT (human keratinocytes). Clinically established anticancer agents, cisplatin and doxorubicin, were employed as comparator reference drugs, and the cytotoxicity data obtained are compiled in Table 1. The IC_50_ values were used to express compound activity, and the selectivity index (SI) was determined as the ratio of effects on normal versus cancerous cells. The tested 1,2,3-triazole–dipyridothiazine hybrids exhibited distinct cytotoxic profiles across the evaluated cancer cell lines, reflecting differential selectivity and potency. Among them, **TDT3b**, a hybrid of 2,7-diazaphenothiazine with a 1,2,3-triazole bearing a *p*-trifluoromethylphenyl substituent, demonstrated the highest activity against A549 lung cancer cells (IC_50_ = 0.14 μM), comparable to the reference drug doxorubicin, and displayed a high selectivity index. This compound also showed notable cytotoxicity toward MDA-MB-231 breast cancer cells (IC_50_ = 13.7 μM), while exhibiting negligible activity against MCF-7 breast cancer, C32 melanoma, and SNB-19 glioblastoma cells, highlighting its selective targeting of specific tumor types. Compound **TDT2b**, incorporating a 1,8-diazaphenothiazine core linked to a 1,2,3-triazole with a *p*-trifluoromethylphenyl substituent, displayed a complementary pattern of activity, showing potent effects against SNB-19 glioma (IC_50_ = 7.2 μM), MCF-7 breast cancer (IC_50_ = 7.74 μM), and C32 melanoma (IC_50_ = 10.2 μM), consistent with selective targeting of rapidly proliferating or metabolically active cells. In contrast, moderate activity was observed against A549 lung cancer and MDA-MB-231 breast cancer cells (IC_50_ = 31.9–51.7 μM), suggesting differences in cellular uptake, target engagement, or pathway susceptibility. Notably, **TDT2b** exhibited favorable selectivity indices, indicating a preferential cytotoxic effect on malignant cells and supporting its potential for further mechanistic investigation. Within the same series, **TDT1a**, comprising a 1,6-diazaphenothiazine core conjugated to a 1,2,3-triazole bearing a *p*-fluorophenyl substituent, showed moderate activity against A549 lung cancer cells (IC_50_ = 38 μM). In contrast, **TDT4a** displayed pronounced cytotoxicity, with IC_50_ values ranging from 1.98 to 2.45 μM toward MDA-MB231 breast cancer and A-549 lung cancer cell lines. The selective activity of **TDT1a** toward lung cancer cells suggests some degree of tumor-specific targeting. This differential cytotoxicity may involve partial activation of mitochondrial apoptotic pathways as well as additional modulatory effects, highlighting the relevance of these compounds for further mechanistic studies despite moderate potency. Collectively, these findings underscore the critical role of both the heterocyclic core and the nature of triazole substituents in modulating anticancer potency and cell-type selectivity within this series.

#### Apoptosis Assessment via Transcriptional Analysis of H3, TP53, CDKN1A, BCL-2, and BAX

Compound **TDT2b** was identified as the most promising hybrid based on its cytotoxic and selectivity profiles and was therefore selected for detailed mechanistic studies using RT-qPCR. This analysis enabled the evaluation of transcriptional changes in key genes associated with tumor biology, including those regulating cell proliferation (*H3*), cell cycle control and DNA damage response (*TP53* and *CDKN1A*), as well as the intrinsic mitochondrial apoptotic pathway (*BCL-2* and *BAX*) [48,49]. The observed gene expression patterns provide insight into the molecular mechanisms underlying the anticancer activity of compound **TDT2b**. The results obtained for three cancer cell lines are summarized in Table 2.

The gene encoding histone *H3* plays a central role in regulating chromatin organization and the transcriptional control of genetic information [29,48]. Treatment with the hybrid compound **TDT2b** resulted in a significant reduction of *H3* mRNA levels across all three tested cancer cell lines, suggesting that its anticancer effects may involve alterations in chromatin structure and epigenetic regulation of gene expression. Tumor protein p53, encoded by *TP53*, is a key mediator of the cellular stress response, orchestrating DNA damage repair, cell cycle arrest, and apoptosis [48,49]. The significant downregulation of *TP53* observed following **TDT2b** treatment in all examined cell lines may indicate a compound-induced modulation of p53-dependent stress signaling, potentially triggering early apoptotic events or activating alternative, p53-independent cell death pathways, possibly linked to mitochondrial dysfunction. The cell cycle inhibitor *CDKN1A* (p21), a critical downstream target of p53, regulates DNA damage responses as well as normal cell cycle progression [48]. **TDT2b** induced a marked upregulation of *CDKN1A* in SNB-19 glioblastoma cells, whereas it caused significant downregulation in C32 melanoma and MCF-7 breast cancer cells. These contrasting effects suggest the activation of distinct, cell line–specific mechanistic pathways, potentially reflecting differences in p53 signaling status and cell cycle regulation. The compound also modulated key regulators of the intrinsic apoptotic pathway, *BCL-2* and *BAX*. In SNB-19 cells, both genes were upregulated, whereas in C32 cells, they were downregulated. In MCF-7 cells, a differential pattern was observed, with *BCL-2* decreasing and *BAX* increasing. These cell type-dependent changes indicate that **TDT2b** may shift the balance between pro- and antiapoptotic signals in a context-specific manner, promoting mitochondrial apoptosis and modulating survival pathways through its effects on HDAC6 [50]. Taken together, these findings suggest that the anticancer activity of **TDT2b** involves a multifaceted mechanism, including chromatin remodeling, modulation of p53-related stress responses, and regulation of cell cycle checkpoints, which may collectively contribute to its selective cytotoxicity toward cancer cells.

### 3.3. Molecular Docking

Building on the observed cytotoxicity and gene expression results, molecular docking analyses were performed for **TDT2b** and **TDT3b** with human histone deacetylase 6 (HDAC6), a key regulator of cytoplasmic and nuclear protein acetylation previously identified as a promising anticancer target [23,50]. HDAC6 modulates multiple cellular processes, including chromatin remodeling, protein stability, stress response, and the mitochondrial apoptotic pathway. By assessing the potential binding interactions of these hybrids with HDAC6, the study aimed to elucidate whether their anticancer activity might involve inhibition of this enzyme, thereby contributing to epigenetic regulation, disruption of cell survival signaling, and the induction of apoptosis in cancer cells. The docking results provide mechanistic insights supporting the hypothesis that **TDT2b** and **TDT3b** exert their effects, at least in part, through HDAC6-mediated modulation of chromatin structure and apoptotic pathways.

The molecular dynamics (MD) results used in this study originate from our previous work, where the full simulation protocol and analysis are reported [48]. For the purposes of the present study, the structure corresponding to the last frame of the MD trajectory was extracted and employed as the input conformation for the docking calculations. For this study, we selected two compounds: **TDT2b** and **TDT3b** (both compounds with promising IC_50_ values). Table 3 summarizes the most important docking results.

Both compounds present similar docking results. The average binding affinity values were −7.999 kcal/mol (**TDT2b**), −8.097 kcal/mol (**TDT3b** Low values of the INTER energy parameter were also recorded; average values: −10.063 kcal/mol and −10.099 kcal/mol, respectively).

**Figure 3:**
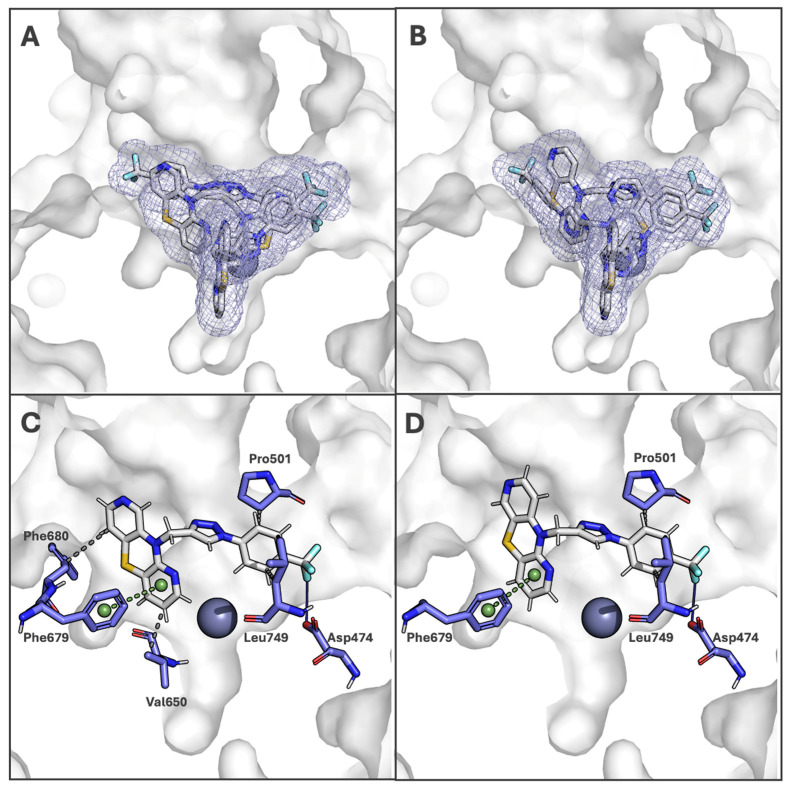
(**A**,**B**)—the space in which the compounds docked (light blue mesh). The graphics also show the position of the most diverse poses in relation to RMSD values. (**B**–**D**)—interactions between analyzed compounds (**TDT2b**, **TDT3b** respectively) in the active site pocket. Interactions are marked as follows: hydrogen bonds—dark blue lines; hydrophobic interactions—gray dotted lines; π-stacking interactions—green dotted lines; cation-π interactions—orange dotted lines.

The ligand internal energies (INTRA) were as follows: for EM1, the average value was −4.439 kcal/mol, and for **TDT3b**, the average value was −0.512 kcal/mol. The INTRA values indicate the absence of unfavorable conformations in all poses of the analyzed ligands. Both compounds dock relatively stably and have a similar binding pocket for all poses (Figure 3A,B). The average RMSD lower bound (LB) values are 3.369 Å (**TDT2b**) and 4.120 Å (**TDT3b**). The upper bound (UB) values are 5.347 Å (**TDT2b**) and 5.950 Å (**TDT3b**) (for more information, see Supplementary Materials Table S1). The best poses (marked as pose 1) were selected for interaction analysis.

Both ligands adopt very similar binding positions in the first pose, with an RMSD of 2.233 Å after alignment. For these poses, a protein–ligand interaction analysis was performed. Among the analyzed triazoles, there is a significantly higher likelihood of interaction: for both compounds, hydrogen bonds with Asp474, π-stacking interactions with Phe679, and hydrophobic interactions with Pro501 and Leu749 were detected. Additionally, **TDT2b** also interacts with Val650 and Phe680 via further hydrophobic contacts (Figure 3C,D). Taking the above into account, it can be assumed that analyses indicate that both compounds exhibit a very similar binding mode to HDAC6. Similar docking positions allowed for the identification of key residues involved in interactions: Asp474, Phe679, Pro501, and Leu749. Additional hydrophobic interactions observed for **TDT2b** may indicate a slightly better fit of this ligand to the binding site. The docking results obtained are consistent with the good biological activity of both compounds.

### 3.4. In Silico Pharmacokinetic Analysis

To evaluate the pharmacokinetic properties and drug-likeness of the synthesized hybrids, preliminary in silico calculations were performed using the SwissADME server [47]. This widely applied computational platform in early-stage drug discovery enables the prediction of key ADME parameters [51,52] and the assessment of drug-likeness based on established criteria, including Lipinski’s Rule of Five and other medicinal chemistry filters [53,54,55]. The analyzed compounds were evaluated with respect to essential molecular descriptors, such as molecular weight, the number of hydrogen bond donors and acceptors, the number of rotatable bonds, molar refractivity, and lipophilicity (Table 4). These parameters were used to determine compliance with Lipinski’s Rule of Five, Ghose’s filter, and Veber’s rule. As the compounds represent structural isomers, no significant differences in molecular descriptors were observed. Minor variations were detected only in lipophilicity, with calculated *logP* values ranging from 3 to 4. All derivatives satisfied the criteria of Lipinski’s and Veber’s rules, indicating favorable drug-likeness characteristics. Overall, the obtained results suggest that the tested hybrids possess suitable physicochemical properties to be considered potential orally active drug candidates.

## 4. Conclusions

Herein, we report the synthesis of novel dipyridothiazine and 1,2,3-triazole hybrids bearing *p*-fluorophenyl and *p*-trifluoromethylphenyl substituents, efficiently obtained via dipolar cycloaddition reactions. The structures of the synthesized compounds were confirmed using spectroscopic techniques, including ^1^H, ^13^C, and ^19^F NMR spectroscopy, as well as high-resolution mass spectrometry (HRMS). The cytotoxic activity of the synthesized derivatives was evaluated using WST-1 and MTT assays against a panel of human cancer cell lines: SNB-19 (glioblastoma), C32 (amelanotic melanoma), A549 (lung carcinoma), MDA-MB-231 and MCF-7 (breast cancer), as well as two normal cell lines, HFF-1 (human fibroblasts) and HaCaT (human keratinocytes). Doxorubicin and cisplatin were used as reference drugs. Selectivity indices were determined to assess cancer cell specificity. Among the tested compounds, the most potent cytotoxic activity was observed for the **TDT2b** and **TDT3b** hybrids composed of 1,8-diazaphenothiazine and 2,7-diazaphenothiazine, respectively, connected to a 1,2,3-triazole ring with a *p*-trifluoromethylphenyl substituent. For the **TDT2b** derivative, gene expression analysis of *H3*, *TP53*, *CDKN1A*, *BCL-2*, and *BAX* was performed using RT-qPCR. The results indicated that the anticancer activity of **TDT2b** involves a multifaceted mechanism, including chromatin remodeling, modulation of p53-dependent stress responses, and regulation of cell cycle checkpoints in the studied cell lines. Based on the cytotoxicity and gene expression data, molecular docking studies were conducted for **TDT2b** and **TDT3b** with human histone deacetylase 6 (HDAC6), a key regulator of cytoplasmic and nuclear protein acetylation and a recognized anticancer target. The docking analyses demonstrated favorable binding interactions for both compounds, supporting their observed biological activity and corroborating the experimental findings. Preliminary in silico evaluation using the SwissADME server indicated that the tested derivatives demonstrate favorable characteristics and may be further considered as candidates for orally active drugs. The presented results provide a biomolecule-centric framework for further optimization and mechanistic validation of dipyridothiazine–triazole hybrids.

## Figures and Tables

**Table 1:** Anticancer activities IC_50_ (μM) of new 1,2,3-triazole and dipyridothiazine hybrids against glioblastoma SNB-19, melanoma C32, breast cancers MCF7, MDA-MB-231, lung cancer A549, and normal cells: NHDF and HaCaT.

No	Anticancer Activity IC_50_ (μM)											
	SNB-19 ^#^		C32 ^#^		MCF-7 ^#^		HFF-1 ^#^	MDA-MB-231 ^$^		A-549 ^$^		HaCaT ^$^
	IC_50_ (μM)	SI	IC_50_ (μM)	SI	IC_50_	SI	IC_50_	IC_50_ (μM)	SI	IC_50_ (μM)	SI	IC_50_
**TDT1a**	>100	1.00	>100	1.00	>100	1.00	>100	46.8 ± 6.85	0.72	38.2 ± 7.24	0.88	33.6 ± 3.79
**TDT1b**	>100	1.00	92 ± 1.55	1.00	>100	1.00	>100	95.4 ± 12.76	1.04	82.2 ± 6.45	1.21	>100
**TDT2a**	97.8 ± 22.46	1.01	>100	1.00	>100	1.00	98 ± 6.66	>100	1.00	>100	1.00	>100
**TDT2b**	7.2 ± 2.76	3.50	10.2 ± 22.7	2.5	7.74 ± 1.22	3.3	25.2 ± 5.33	51.7 ± 3.79	0.99	31.9 ± 6.44	1.61	51.4 ± 6.37
**TDT3a**	89.3 ± 18.76	1.11	>100	1.00	99.4 ± 8.54	1.01	>100	>100	0.82	47.2 ± 12.5	1.74	82.1 ± 5.27
**TDT3b**	>100	1.00	>100	1.00	96.1 ± 7.23	1.02	>100	13.7 ± 1.79	4.17	0.14 ± 0.02	407.85	57.1 ± 6.85
**TDT4a**	62.8 ± 12.76	1.24	89 ± 11.2	0.88	>100	0.78	77.9 ± 9.72	2.45 ± 0.78	2.12	1.98 ± 0.28	2.62	5.2 ± 2.78
**TDT4b**	99.1 ± 9.14	1.01	>100	1.00	>100	1.00	72.1 ± 11.14	>100	0.80	>100	0.80	80.4 ± 3.11
*Doxorubicine*	1.25 ± 0.19	1.92	4.78 ± 1.24	0.50	2.15 ± 1.02	1.12	2.4 ± 0.6	1.6 ± 0.23	0.19	0.2 ± 0.09	1.5	0.3 ± 0.1
*Cisplatin*	1.12 ± 0.87	3.21	3.16 ± 1.98	1.13	0.98 ± 0.18	3.67	3.6 ± 0.9	7.8 ± 0.98	0.81	3.2 ± 1.24	5.08	6.3 ± 0.7

Biological activity studies were conducted by two research teams: the Medical University of Silesia and the Medical University of Warsaw, using the WST-1 and MTT assays, respectively. Results from the WST-1 assay are marked ^#^, and those from the MTT assay are marked ^$^.

**Table 2:** The influence of compound **TDT2b** on expression of genes encoding *H3*, *TP53*, *CDKN1A*, *BCL-2*, and *BAX*.

Gene		SNB-19	C-32	MCF-7
		Number of mRNA Copies/μg Total RNA		
*H3*	control	28769 +/− 1200	32479 +/− 2839	31191 +/− 6115
	TDT2b	11803 +/− 27	4659 +/− 1357	18210 +/− 1824
*TP53*	control	29949 +/−6500	41434 +/− 1127	44080 +/− 7413
	TDT2b	15.121 +/− 1506	4217 +/− 1198	14778 +/− 143
*CDKN1A*	control	122.667 +/− 7434	195908 +/− 14427	234944 +/− 20273
	TDT2b	69303 +/− 17535	27141 +/− 2147	141,072 +/− 2043
*BCL-2*	control	7958 +/− 26	10128 +/− 367	3480 +/− 343
	TDT2b	9326 +/− 1040	3091 +/− 129	3345 +/− 91
*BAX*	control	3749 +/− 271	34443 +/− 764	1927 +/− 336
	TDT2b	8738 +/− 392	3459 +/− 583	5397 +/− 1115
*BCL-2/BAX*	control	2.123	2.940	1.806
	TDT2b	1.067	0.894	0.620

**Table 3:** Energy results for analyzed compounds. The table contains the following energies: binding affinities, ligand-protein interactions (INTER), and ligand internal (INTRA).

Docking Pose	Binding Affinity [kcal/mol]		INTER [kcal/mol]		INTRA [kcal/mol]	
	TDT2b	TDT3b	TDT2b	TDT3b	TDT2b	TDT3b
1	−8.388	−8.455	−10.964	−10.326	−0.261	−0.611
2	−8.142	−8.282	−10.196	−10.286	−0.455	−0.438
3	−8.135	−8.224	−10.139	−10.121	−0.503	−0.532
4	−8.070	−8.107	−10.413	−10.165	−0.149	−0.505
5	−8.036	−8.086	−10.090	−10.045	−0.431	−0.437
6	−7.984	−8.025	−10.179	−10.925	−0.277	−0.483
7	−7.935	−7.970	−9.815	−9.762	−0.581	−0.577
8	−7.819	−7.967	−9.694	−9.925	−0.558	−0.410
9	−7.792	−7.941	−9.649	−9.679	−0.570	−0.625
10	−7.689	−7.917	−9.487	−9.760	−0.605	−0.505
**MEAN**	−7.999	−8.097	−10.063	−10.099	−0.439	−0.512
**SD**	0.203	0.174	0.431	0.367	0.158	0.74

**Table 4:** The molecular descriptor and parameters of Lipinski’s, Ghose’s, and Veber’s rules for investigated compounds calculated using SwisADME server [47].

No	Molecular Mass (g/mol)	H-Bond Acceptors	H-Bond Donors	Rotatable Bonds	Molar Refractivity	LogP_avr_.	P-gp Substrate	Lipinski’s Rules	Ghose’s Rules	Veber’s Rules	Muegge’s Rules
**TDT1a**	376.41	5	0	3	102.9	3.25	+	+	+	+	+
**TDT1b**	426.42	7	0	4	107.94	3.96	+	+	+	+	+
**TDT2a**	376.41	5	0	3	102.9	3.15	+	+	+	+	+
**TDT2b**	426.42	7	0	4	107.94	3.91	+	+	+	+	+
**TDT3a**	376.41	5	0	3	102.9	2.94	+	+	+	+	+
**TDT3b**	426.42	7	0	4	107.94	3.72	+	+	+	+	+
**TDT4a**	376.41	5	0	3	102.9	3.06	+	+	+	+	+
**TDT4b**	426.42	7	0	4	107.94	3.73	+	+	+	+	+

## Data Availability

The original contributions presented in this study are included in the article/Supplementary Materials. Further inquiries can be directed to the corresponding author(s).

## Supplementary Materials

The following supporting information can be downloaded at: https://www.mdpi.com/article/10.3390/biom16030349/s1, Supporting materials on spectral analysis of all new compounds and docking analysis are included. 1. NMR spectra and HR MS of TDT1a. 2. NMR spectra and HR MS of TDT2a. 3. NMR spectra and HR MS of TDT3a. 4. NMR spectra and HR MS of TDT4a. 5. NMR spectra and HR MS of TDT1b. 6. NMR spectra and HR MS of TDT2b. 7. NMR spectra and HR MS of TDT3b. 8. NMR spectra and HR MS of TDT4b. 9. Table S1. RMSD lower bound (LB) and upper bound (UB) results for each docking position.
