# Supplementary material for: New 1,2,3-Triazole and Dipyridothiazine Hybrids—Synthesis, Analysis, Cytotoxicity and Molecular Docking
Source: Biomolecules. 2026 Feb 26;16(3):349. doi: 10.3390/biom16030349 (PMC13023989; doi:10.3390/biom16030349)
Supplement: Supplementary file 1 [file biomolecules-16-00349-s001.zip › biomolecules-4146037-supplementary.pdf]

Supplementary Material

Content

1. NMR spectra and HR MS of **TDT1a**
2. NMR spectra and HR MS of **TDT2a**
3. NMR spectra and HR MS of **TDT3a**
4. NMR spectra and HR MS of **TDT4a**
5. NMR spectra and HR MS of **TDT1b**
6. NMR spectra and HR MS of **TDT2b**
7. NMR spectra and HR MS of **TDT3b**
8. NMR spectra and HR MS of **TDT4b**
9. **Table S1**. RMSD lower bound (LB) and upper bound (UB) results for each docking position

NMR spectra and HR MS of **TDT1a**

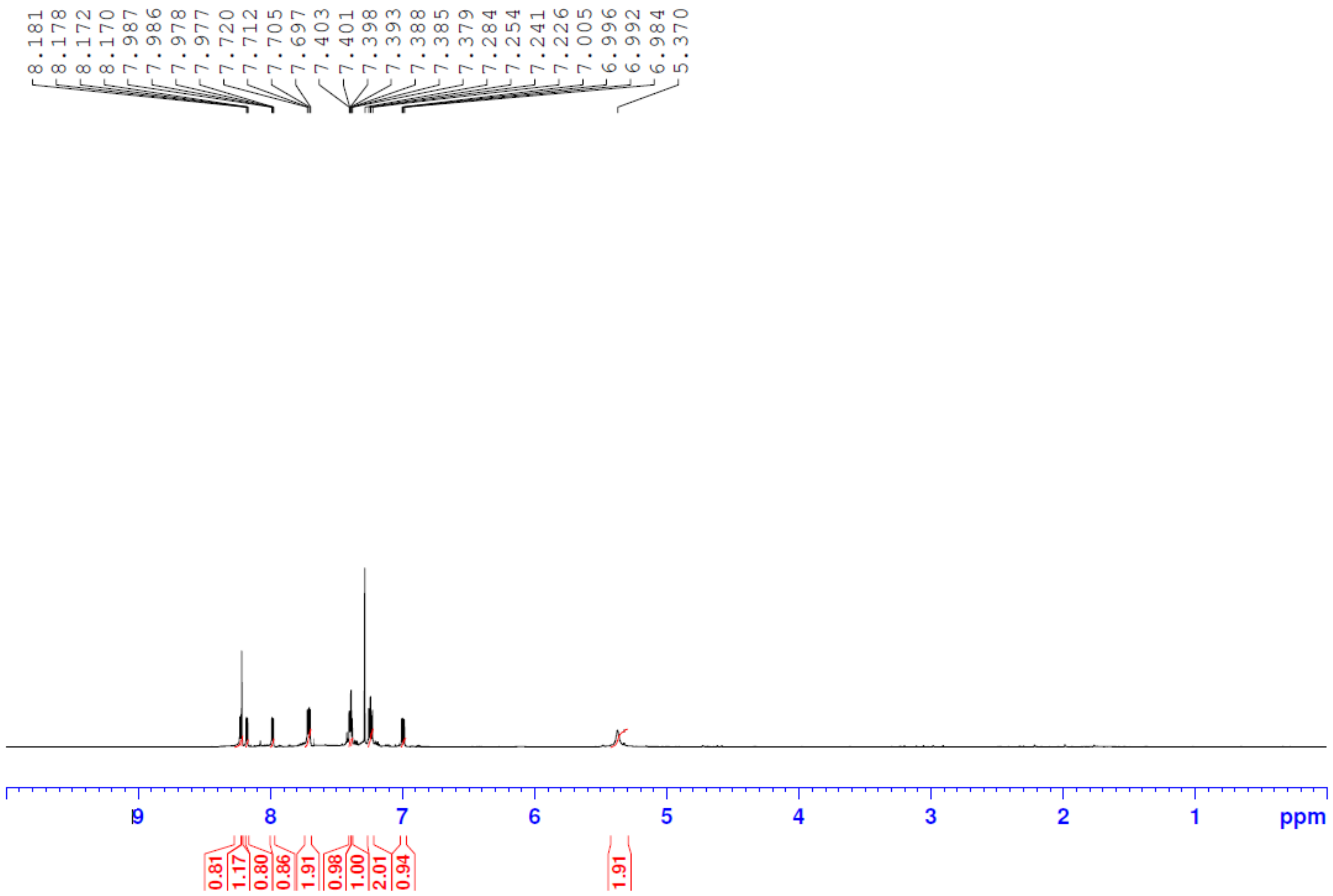

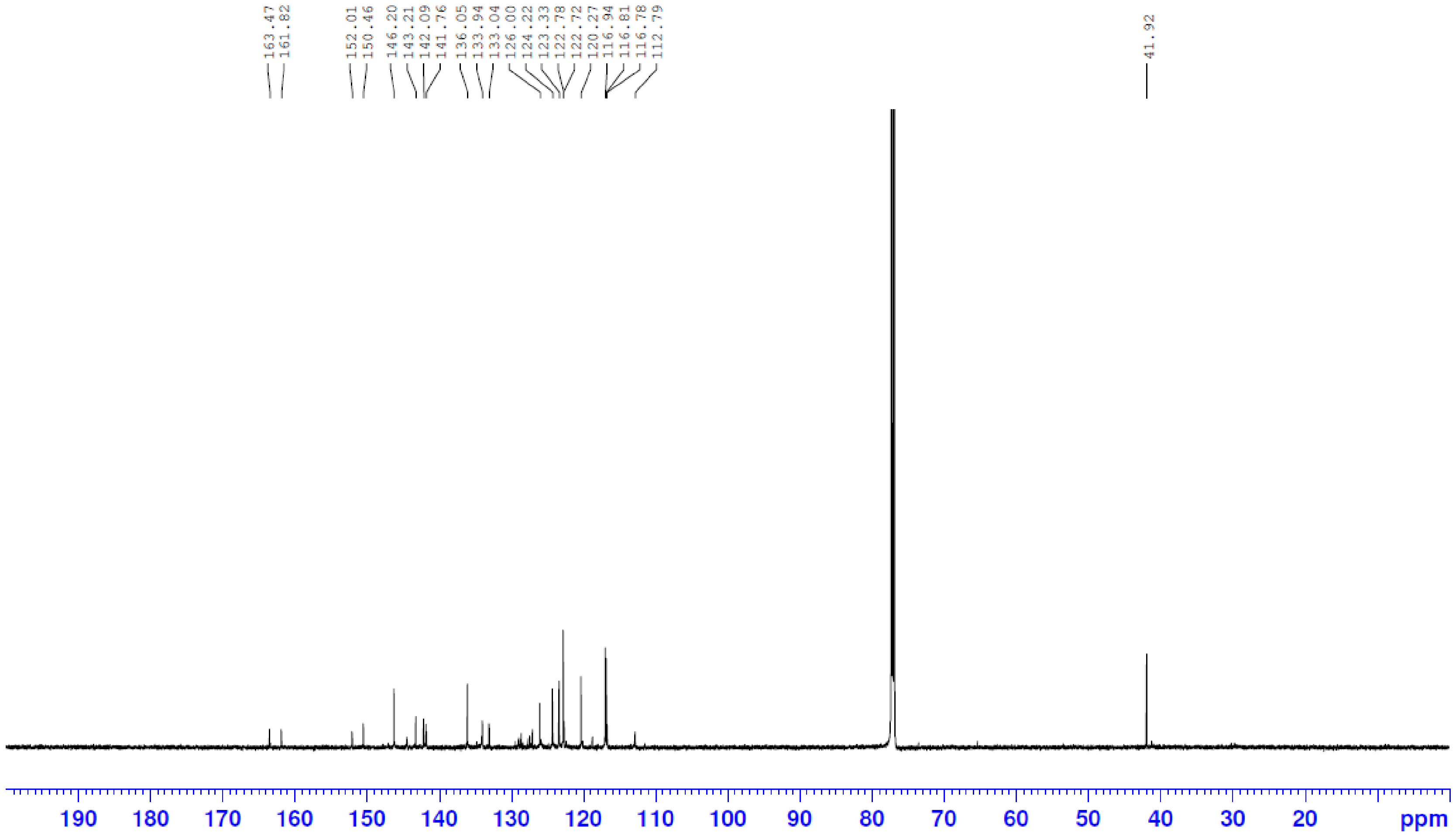
163.47
161.82
152.01
150.46
146.20
143.21
142.09
141.76
136.05
133.94
133.04
126.00
124.22
123.33
122.78
122.72
120.27
116.94
116.81
116.78
112.79
41.92
190
180
170
160
150
140
130
120
110
100
90
80
70
60
50
40
30
20
ppm

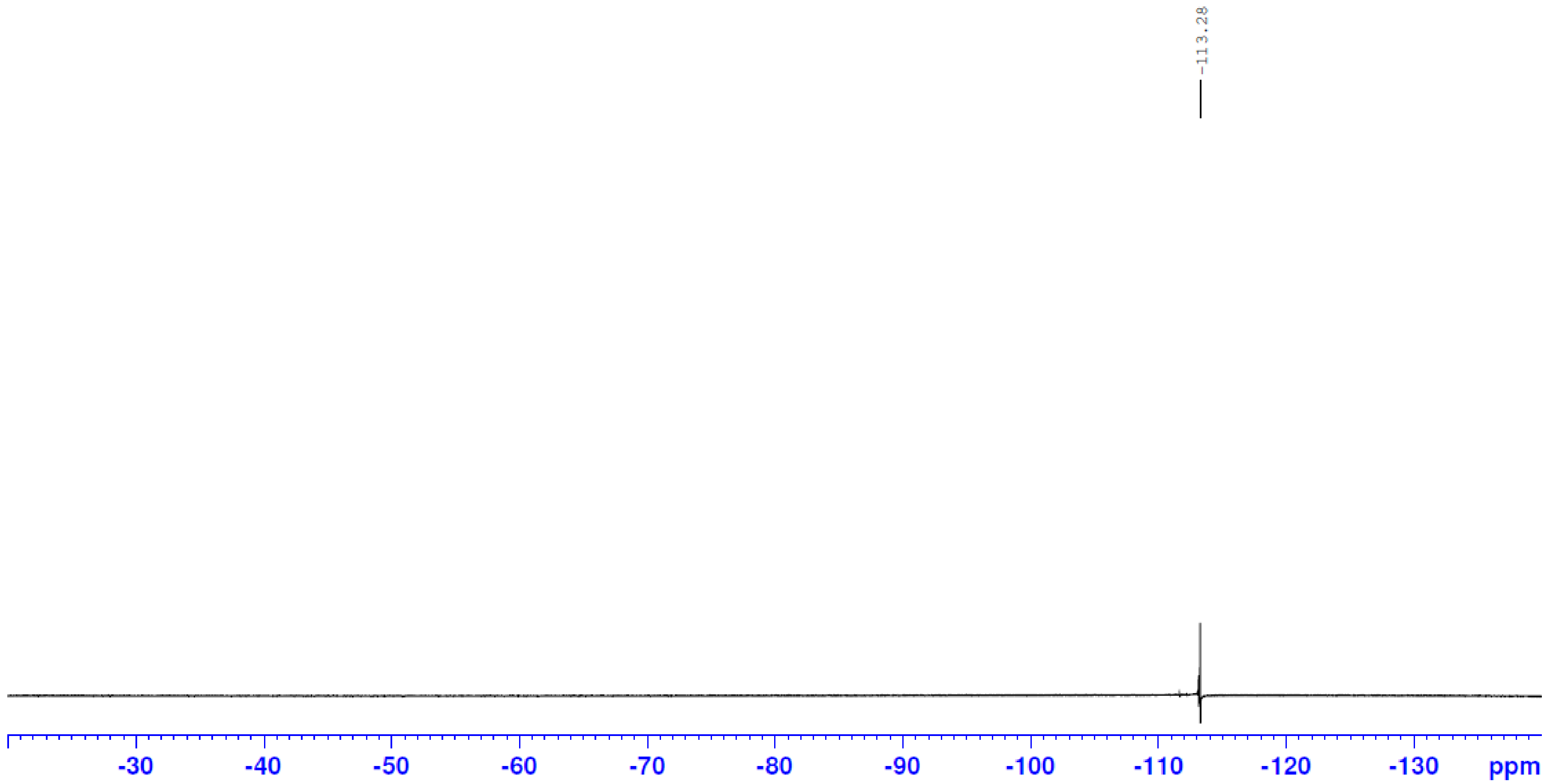
-113.28
-30
-40
-50
-60
-70
-80
-90
-100
-110
-120
-130
ppm

Acquisition Parameter

Source Type	ESI	Ion Polarity	Positive	Set Nebulizer	0.3 Bar
Focus	Active	Set Capillary	4000 V	Set Dry Heater	200 °C
Scan Begin	100 m/z	Set End Plate Offset	-500 V	Set Dry Gas	3.0 l/min
Scan End	1000 m/z	Set Charging Voltage	2000 V	Set Divert Valve	Source
		Set Corona	0 nA	Set APCI Heater	0 °C

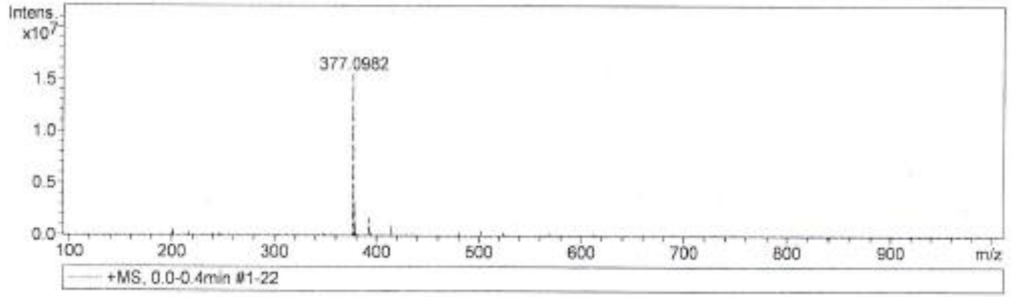

#	m/z	Res.	S/N	I	I %	FWHM
1	377.0982	35869	58852.9	15498033	100.0	0.0105

NMR spectra and HR MS of **TDT2a**

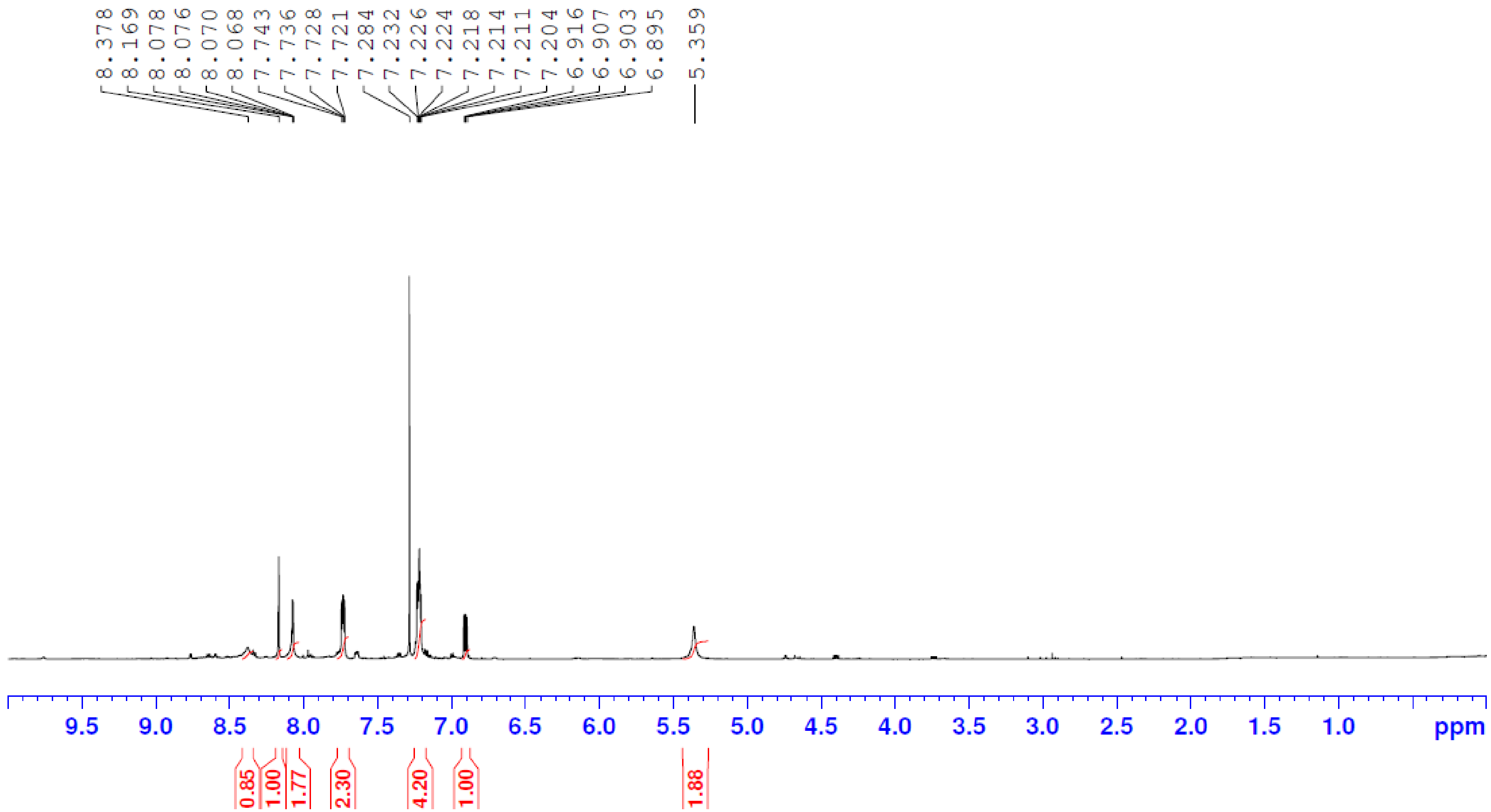

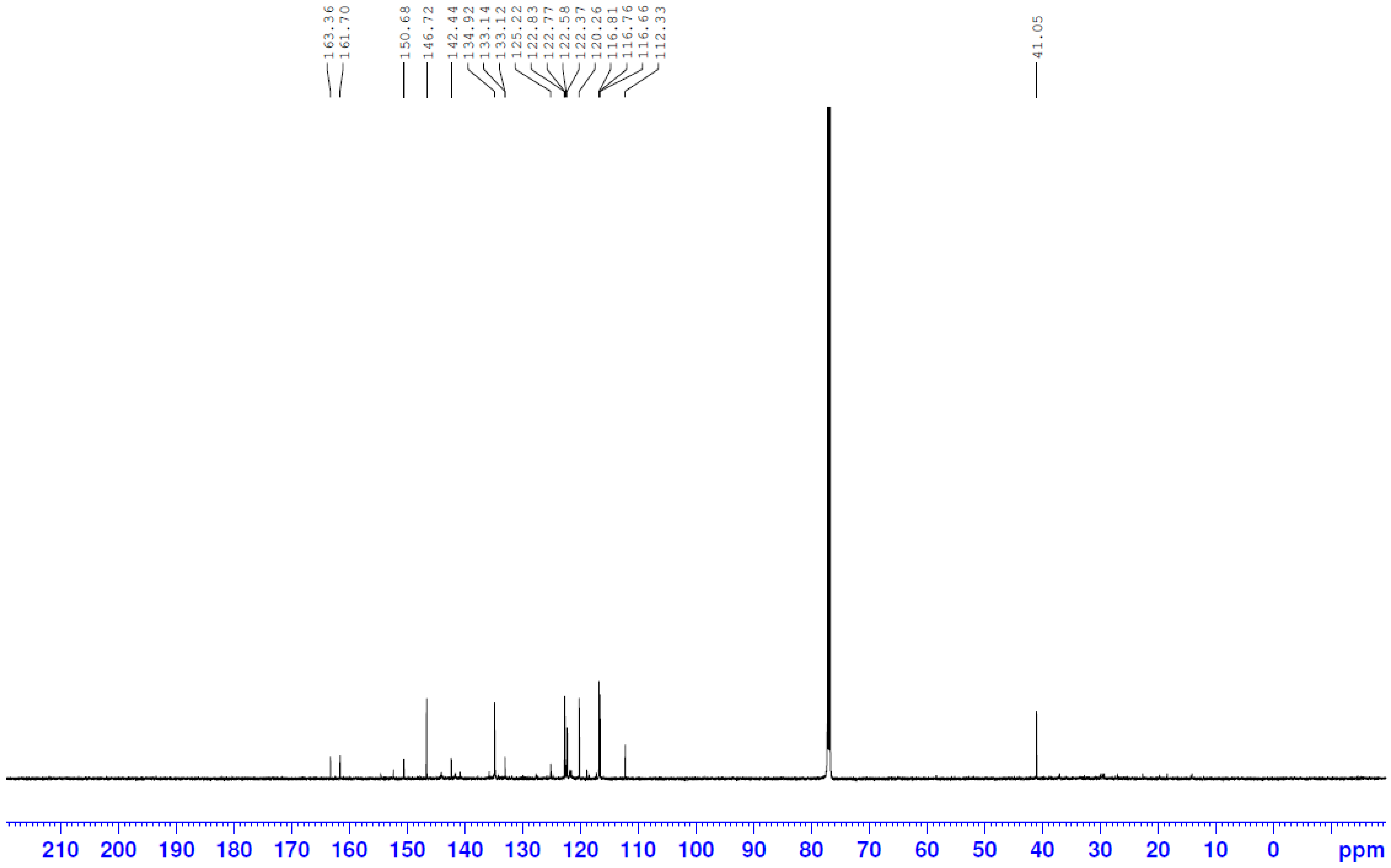
163.36
161.70
150.68
146.72
142.44
134.92
133.14
133.12
125.22
122.83
122.77
122.58
122.37
120.26
116.81
116.76
116.66
112.33
41.05
210 200 190 180 170 160 150 140 130 120 110 100 90 80 70 60 50 40 30 20 10 0 ppm

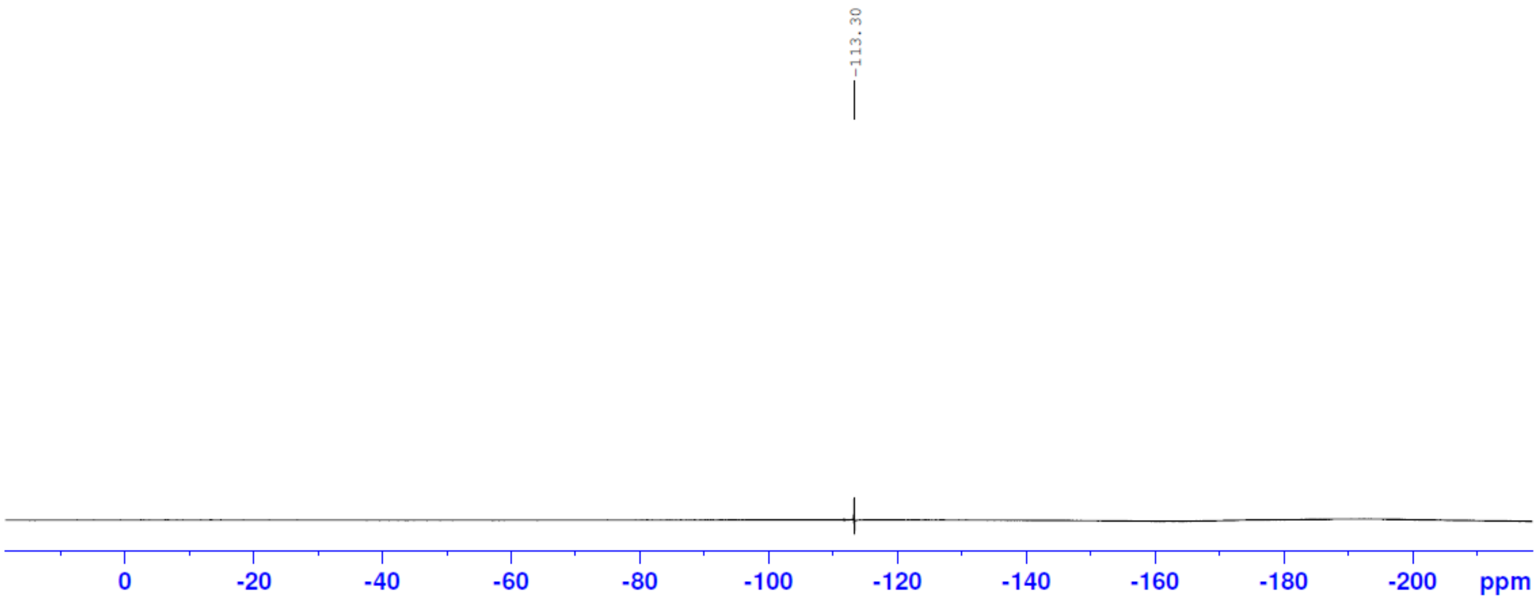
-113.30
0
-20
-40
-60
-80
-100
-120
-140
-160
-180
-200
ppm

Acquisition Parameter

Source Type	ESI	Ion Polarity	Positive	Set Nebulizer	0.3 Bar
Focus	Active	Set Capillary	4000 V	Set Dry Heater	200 °C
Scan Begin	100 m/z	Set End Plate Offset	-500 V	Set Dry Gas	3.0 l/min
Scan End	1000 m/z	Set Charging Voltage	2000 V	Set Divert Valve	Source
		Set Corona	0 nA	Set APCI Heater	0 °C

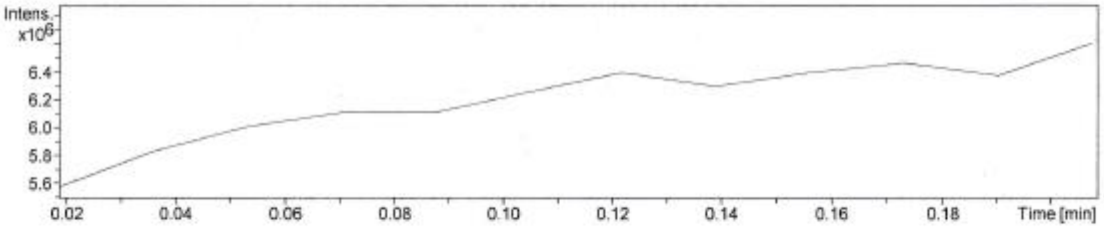

+MS, 0.1-0.2min #4-11

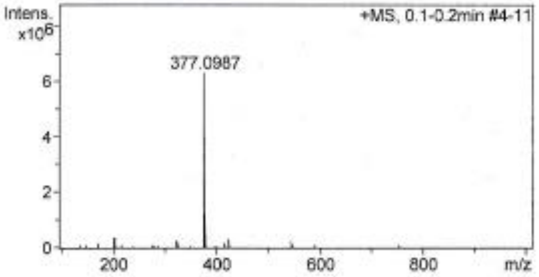

#	m/z	Res.	S/N	I	I %	FWHM
1	377.0987	22160	57115.3	6297122	100.0	0.0170

NMR spectra and HR MS of **TDT3a**

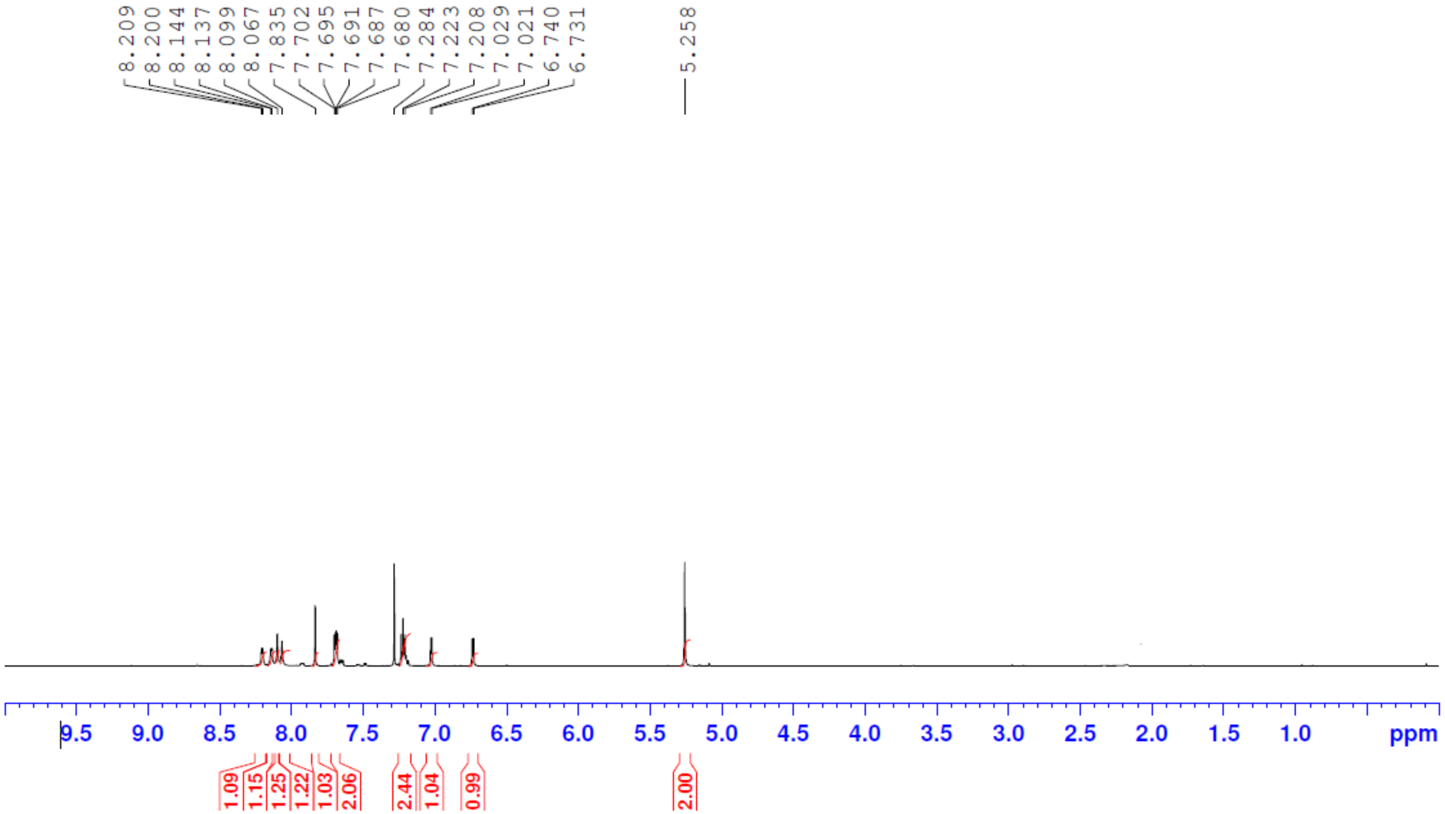

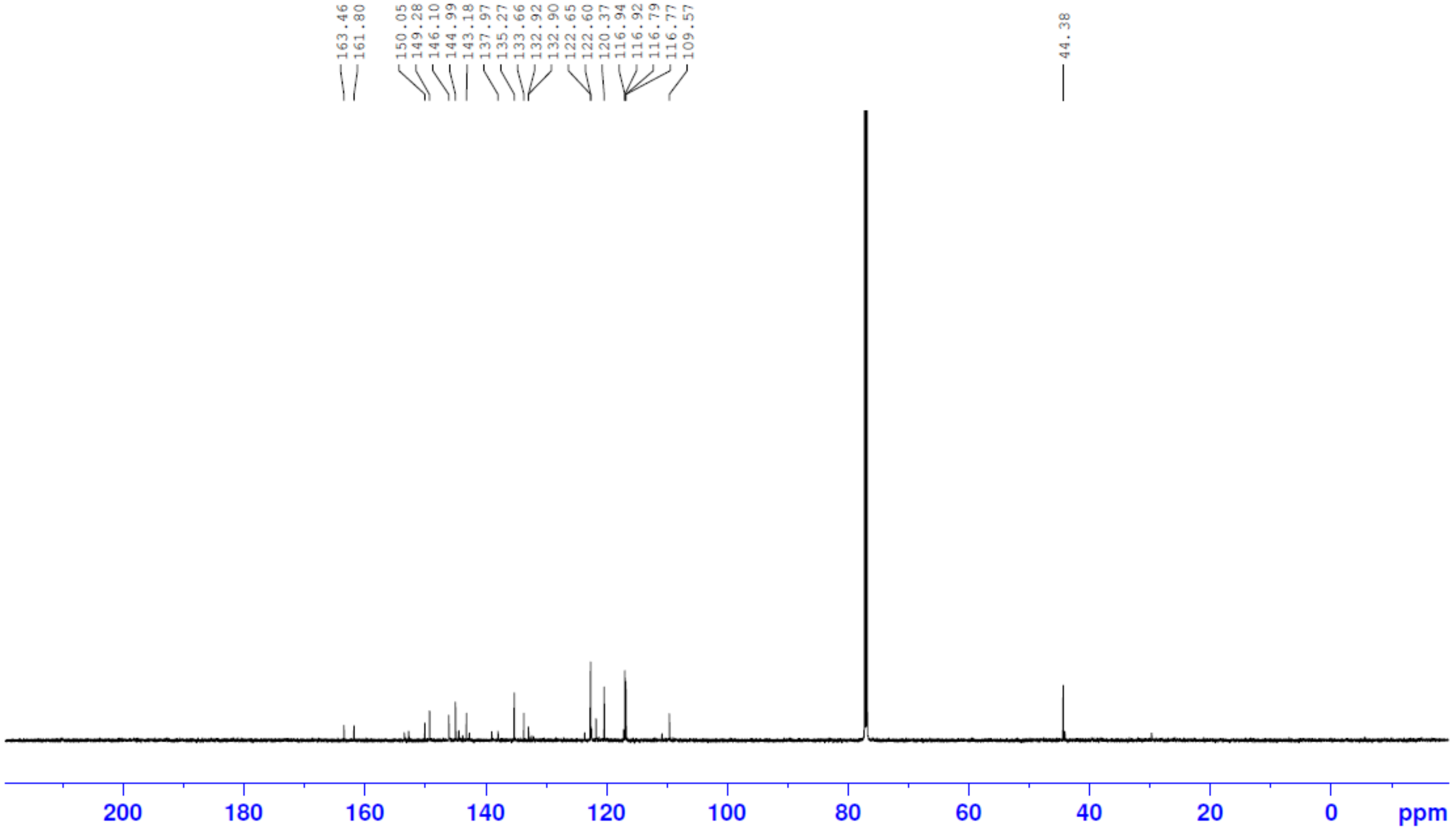
163.46
161.80
150.05
149.28
146.10
144.99
143.18
137.97
135.27
133.66
132.92
132.90
122.65
122.60
120.37
116.94
116.92
116.79
116.77
109.57
44.38
200
180
160
140
120
100
80
60
40
20
0
ppm

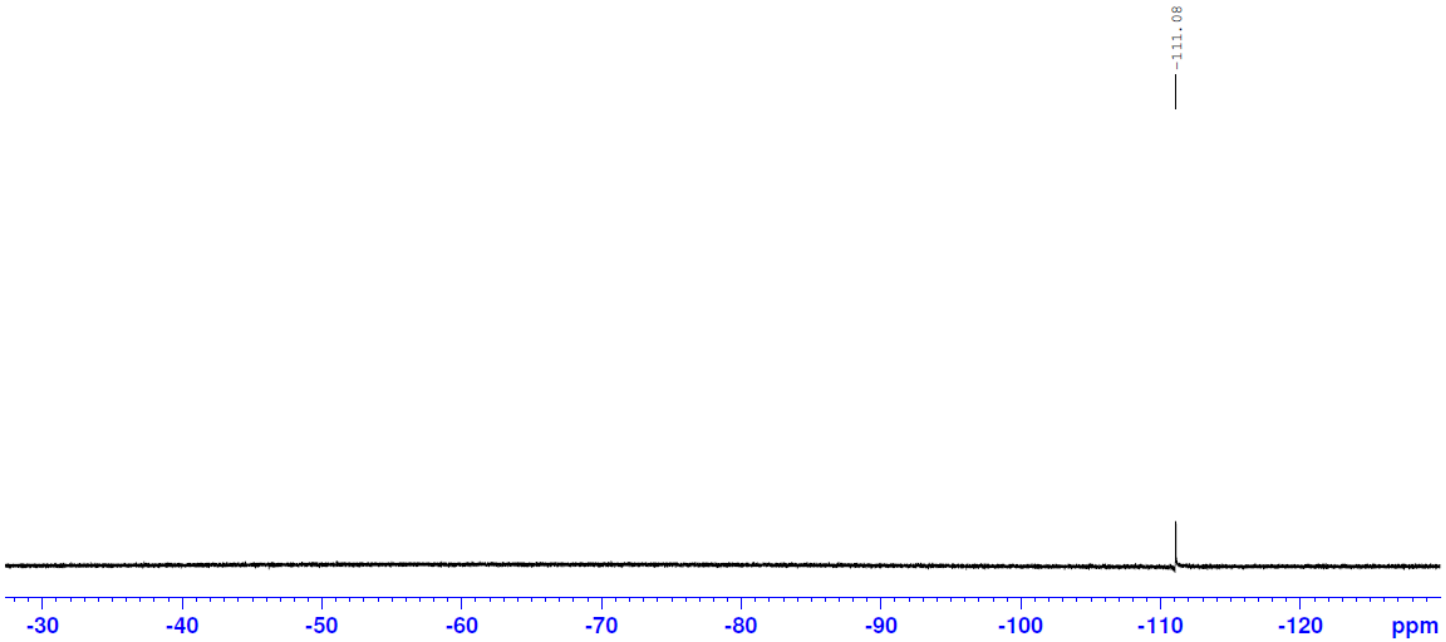

-111.08
-30
-40
-50
-60
-70
-80
-90
-100
-110
-120
ppm

Acquisition Parameter

Source Type	ESI	Ion Polarity	Positive	Set Nebulizer	0.4 Bar
Focus	Active	Set Capillary	4500 V	Set Dry Heater	180 °C
Scan Begin	50 m/z	Set End Plate Offset	-500 V	Set Dry Gas	4.0 l/min
Scan End	1000 m/z	Set Charging Voltage	2000 V	Set Divert Valve	Source
		Set Corona	0 nA	Set APCI Heater	0 °C

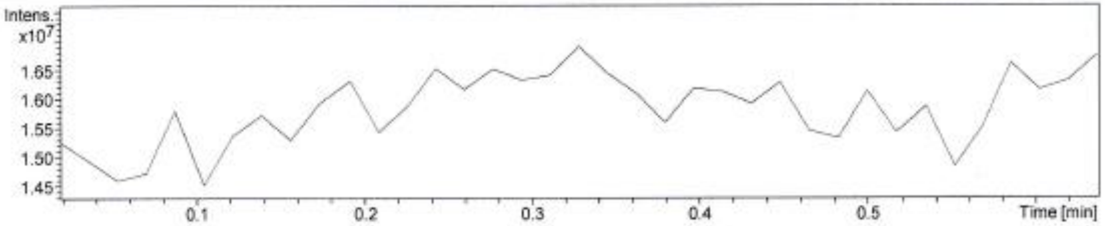

+MS, 0.1-0.6min #7-36

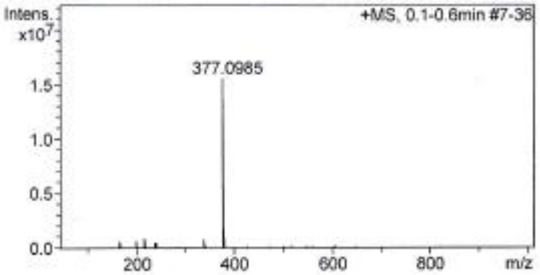

#	m/z	Res.	S/N	I	I %	FWHM
1	377.0985	33147	331004.0	15977968	100.0	0.0072

NMR spectra and HR MS of **TDT4a**

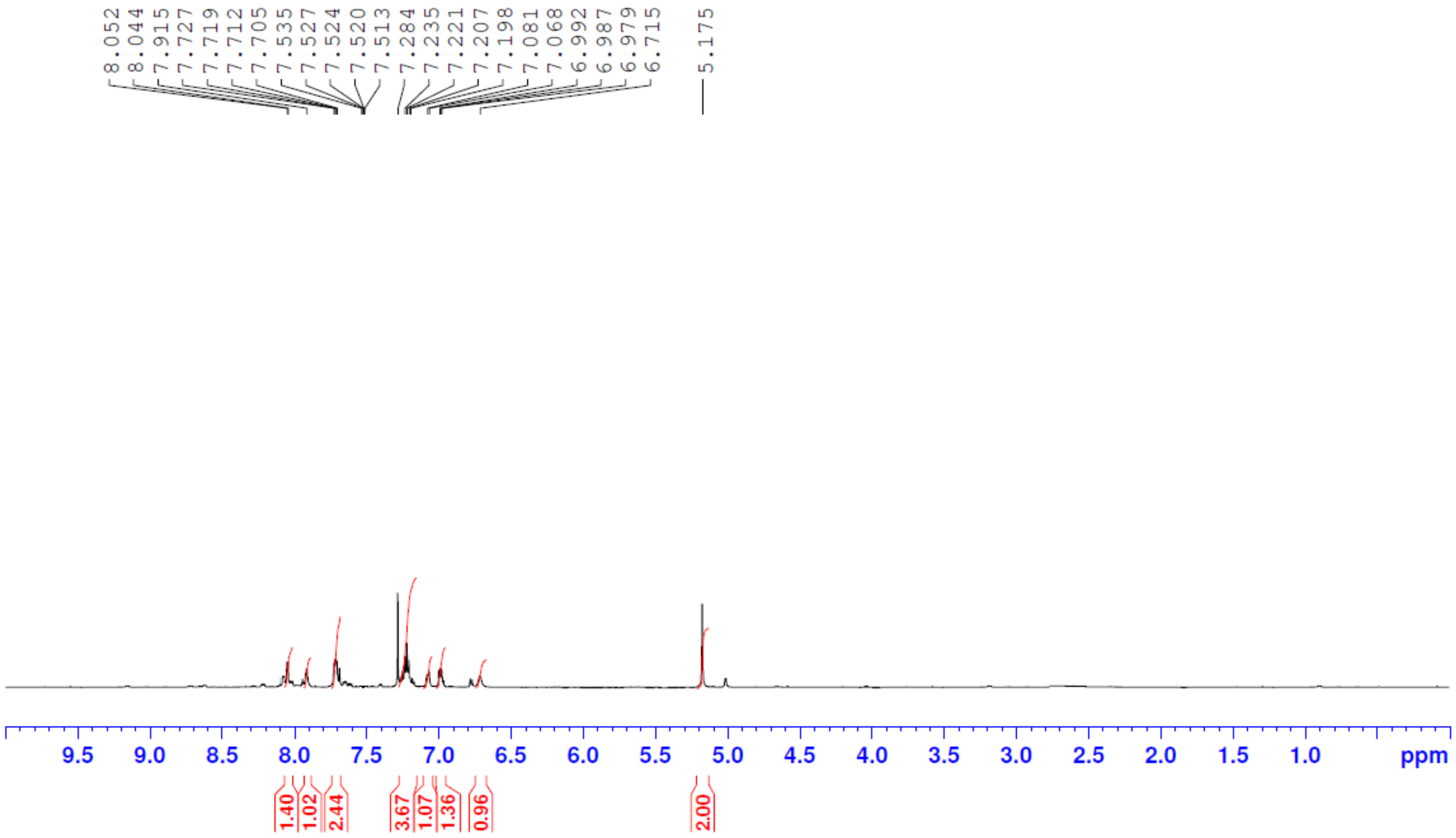

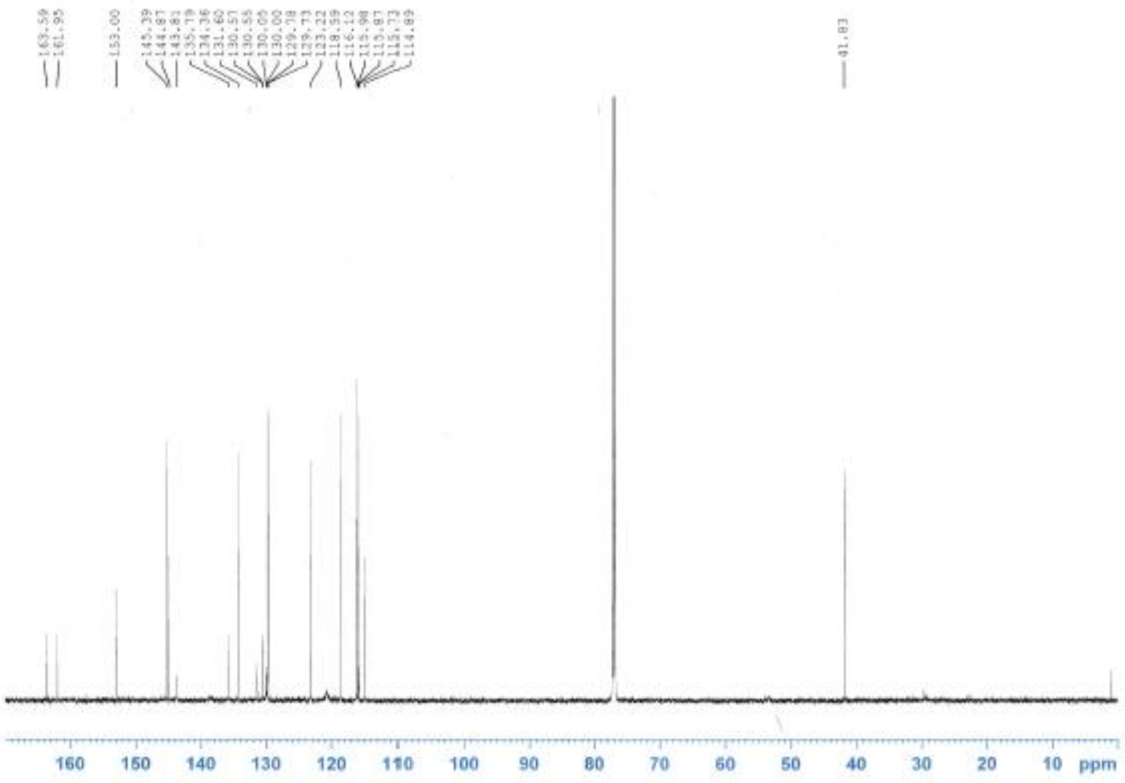
163.59
161.95
153.00
145.39
144.87
143.81
135.79
134.36
131.60
130.57
130.55
130.05
130.00
129.78
129.73
123.22
118.59
116.12
115.98
115.87
115.73
114.89
41.83
160 150 140 130 120 110 100 90 80 70 60 50 40 30 20 10 ppm

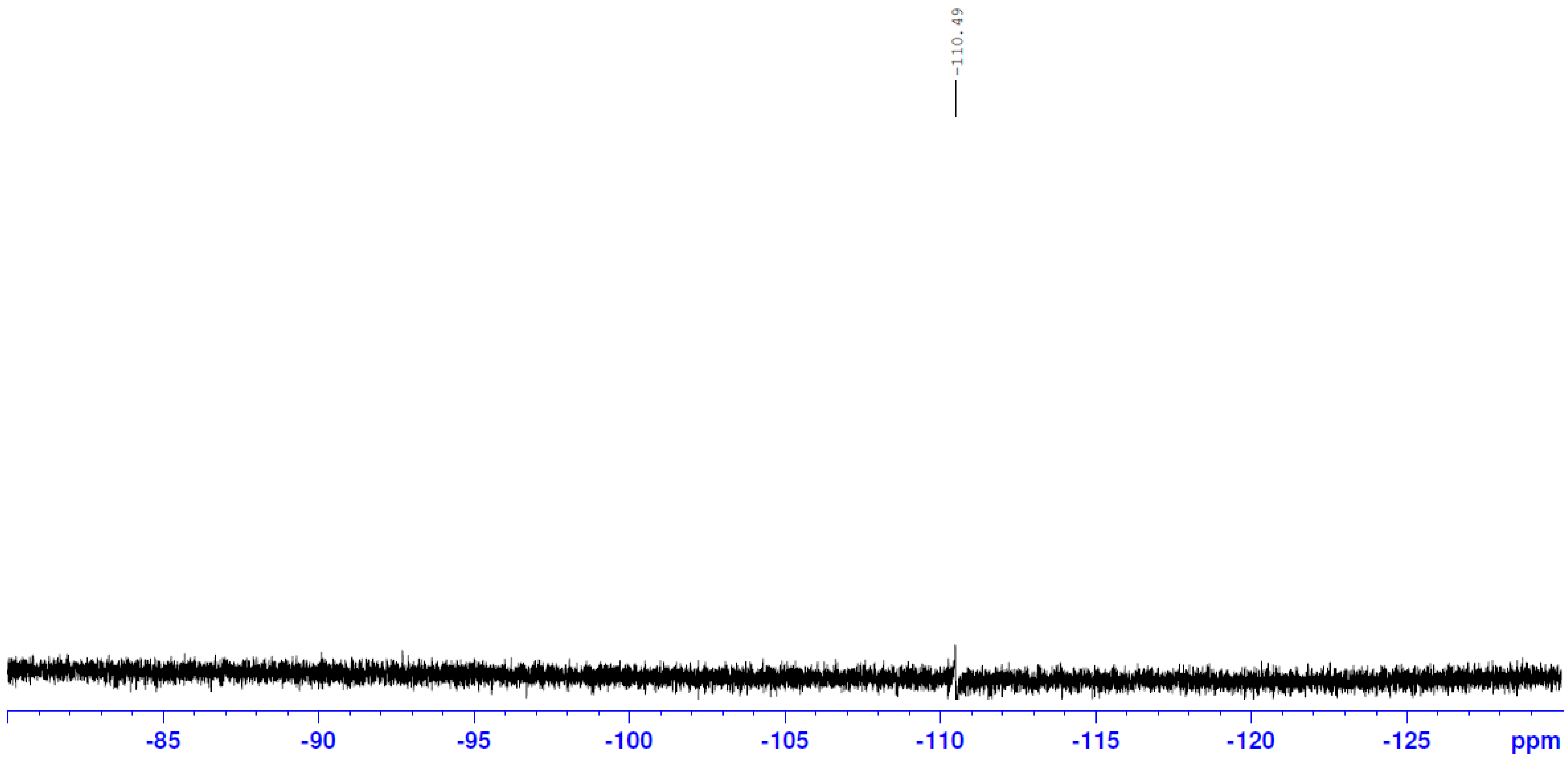
-110.49
-85
-90
-95
-100
-105
-110
-115
-120
-125
ppm

Acquisition Parameter

Source Type	ESI	Ion Polarity	Positive	Set Nebulizer	0.4 Bar
Focus	Active	Set Capillary	4500 V	Set Dry Heater	180 °C
Scan Begin	50 m/z	Set End Plate Offset	-500 V	Set Dry Gas	4.0 l/min
Scan End	1000 m/z	Set Charging Voltage	2000 V	Set Divert Valve	Source
		Set Corona	0 nA	Set APCI Heater	0 °C

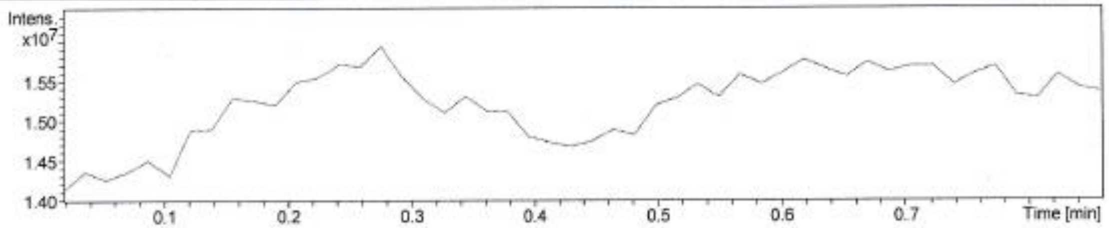

+MS, 0.6-0.8min #35-49

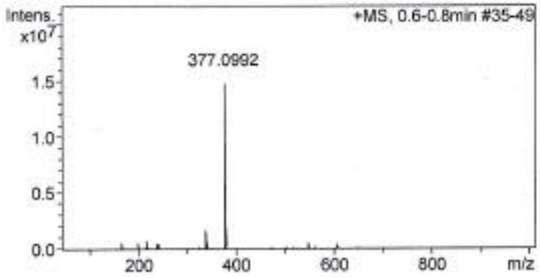

#	m/z	Res.	S/N	I	I %	FWHM
1	377.0992	39731	59469.4	6883279	100.0	0.0071

NMR spectra and HR MS of **TDT1b**

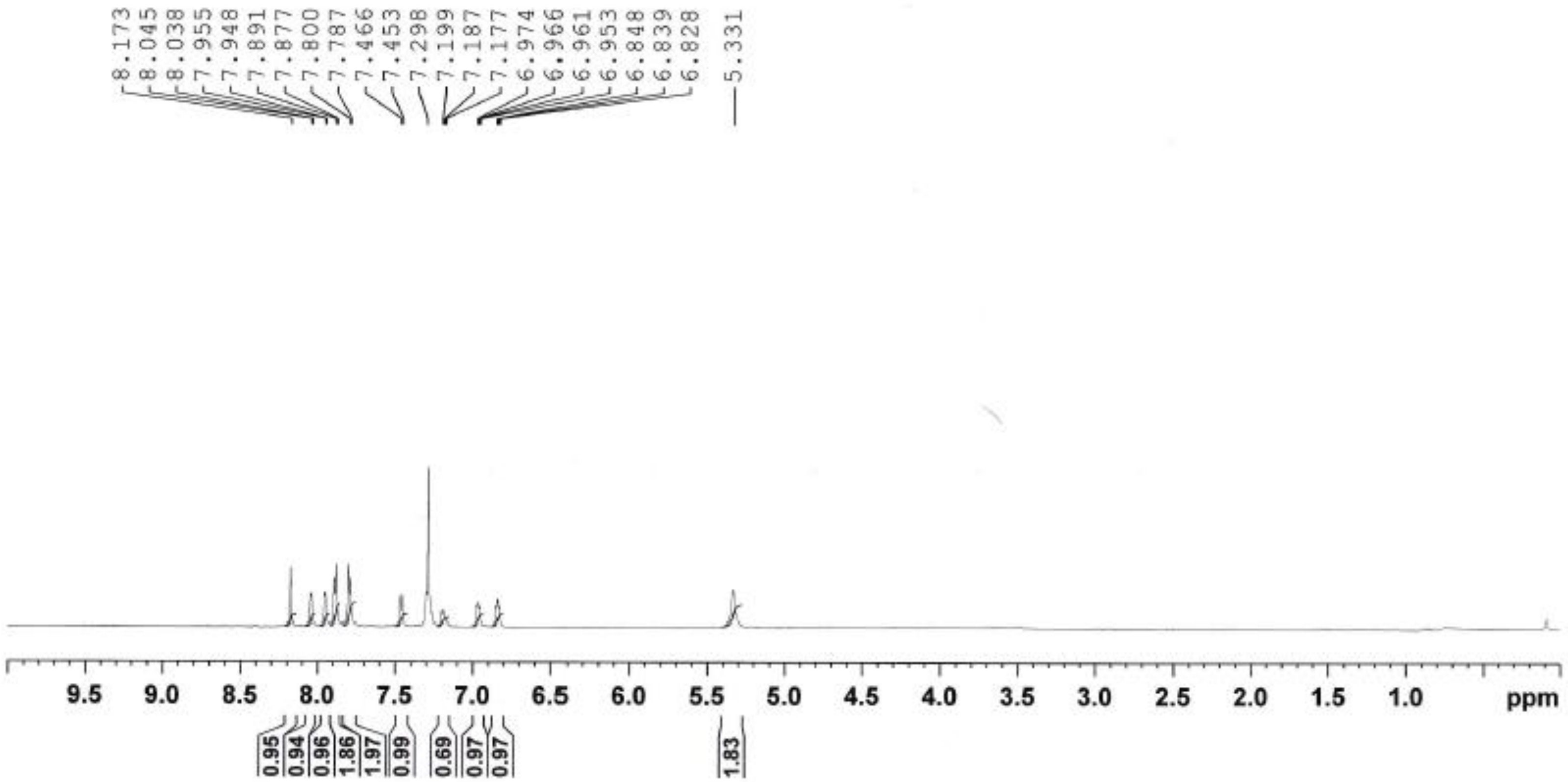

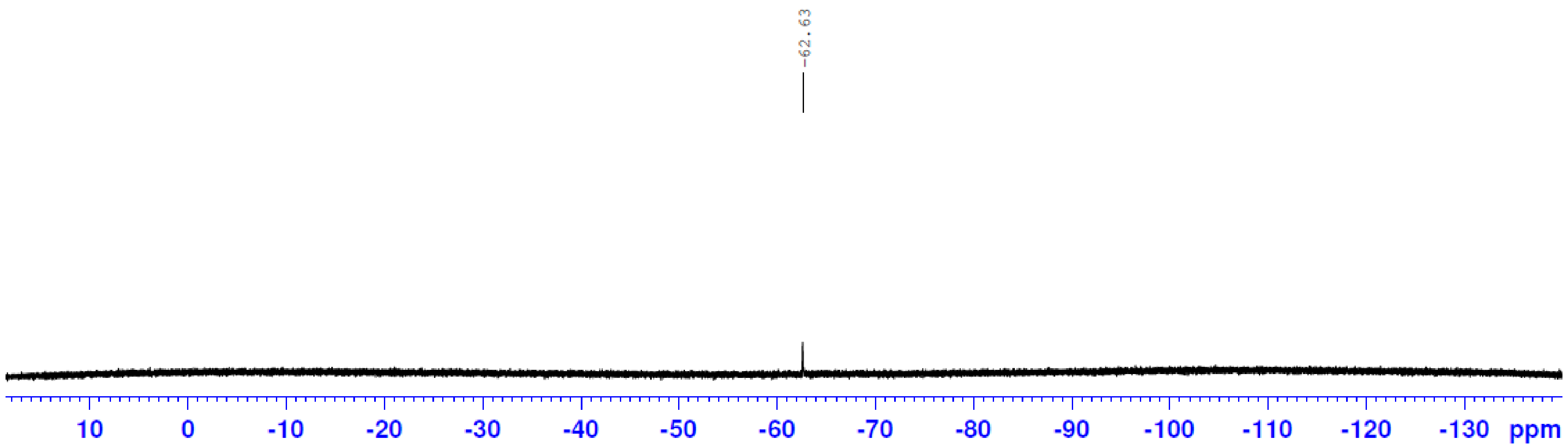
-62.63
10
0
-10
-20
-30
-40
-50
-60
-70
-80
-90
-100
-110
-120
-130
ppm

Acquisition Parameter

Source Type	ESI	Ion Polarity	Positive	Set Nebulizer	0.3 Bar
Focus	Active	Set Capillary	4000 V	Set Dry Heater	240 °C
Scan Begin	100 m/z	Set End Plate Offset	-500 V	Set Dry Gas	4.0 l/min
Scan End	1000 m/z	Set Charging Voltage	2000 V	Set Divert Valve	Source
		Set Corona	0 nA	Set APCI Heater	0 °C

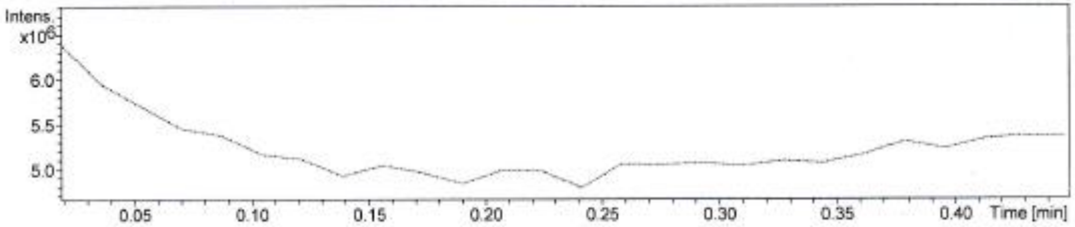

+MS, 0.0-0.4min #2-25

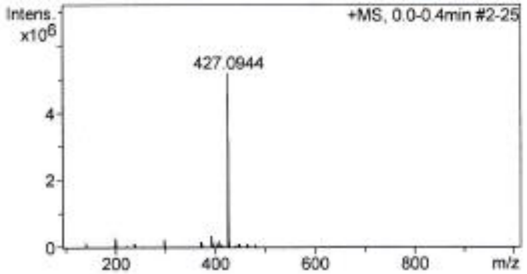

NMR spectra and HR MS of **TDT2b**

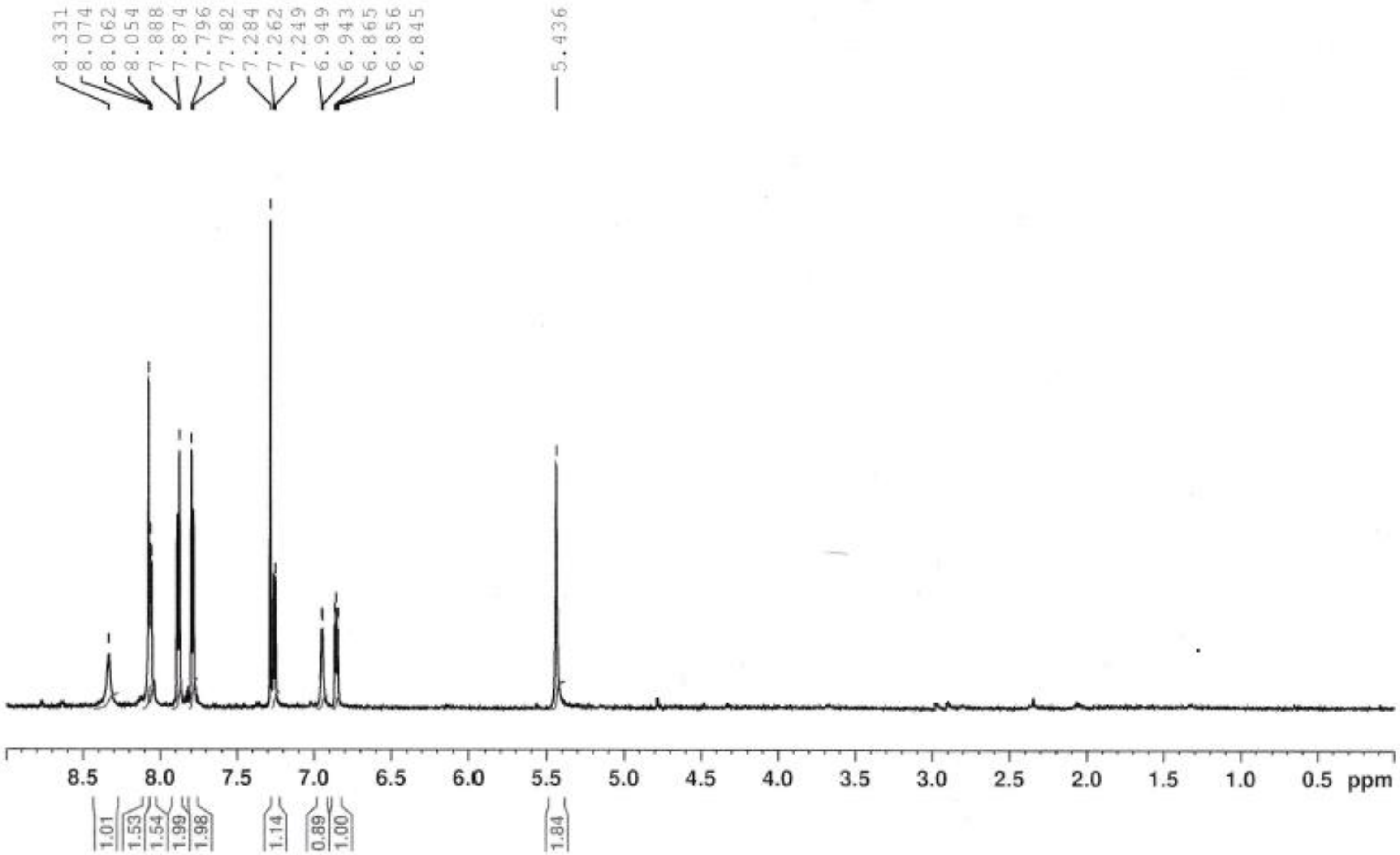

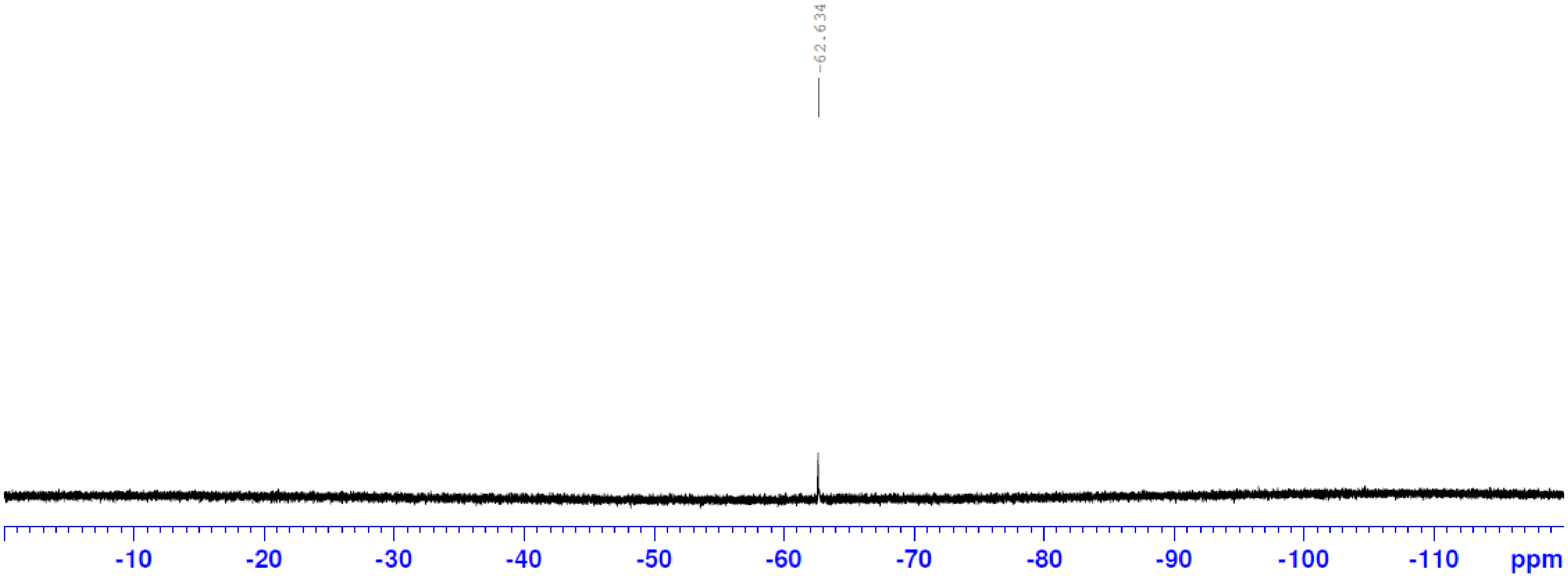

-62.634
-10
-20
-30
-40
-50
-60
-70
-80
-90
-100
-110
ppm

Acquisition Parameter

Source Type	ESI	Ion Polarity	Positive	Set Nebulizer	0.3 Bar
Focus	Active	Set Capillary	4000 V	Set Dry Heater	240 °C
Scan Begin	100 m/z	Set End Plate Offset	-500 V	Set Dry Gas	4.0 l/min
Scan End	1000 m/z	Set Charging Voltage	2000 V	Set Divert Valve	Source
		Set Corona	0 nA	Set APCI Heater	0 °C

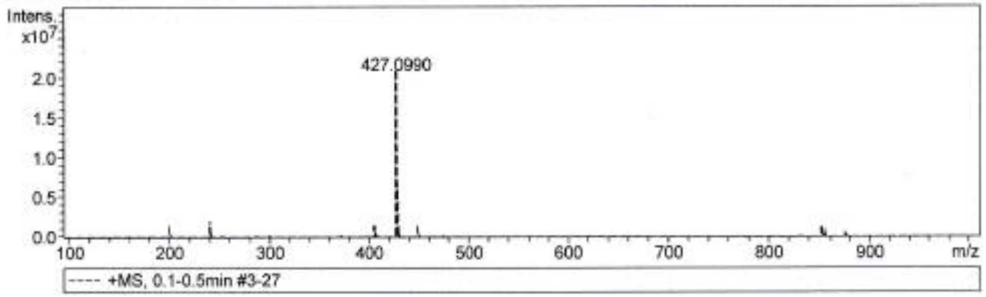

NMR spectra and HR MS of **TDT3b**

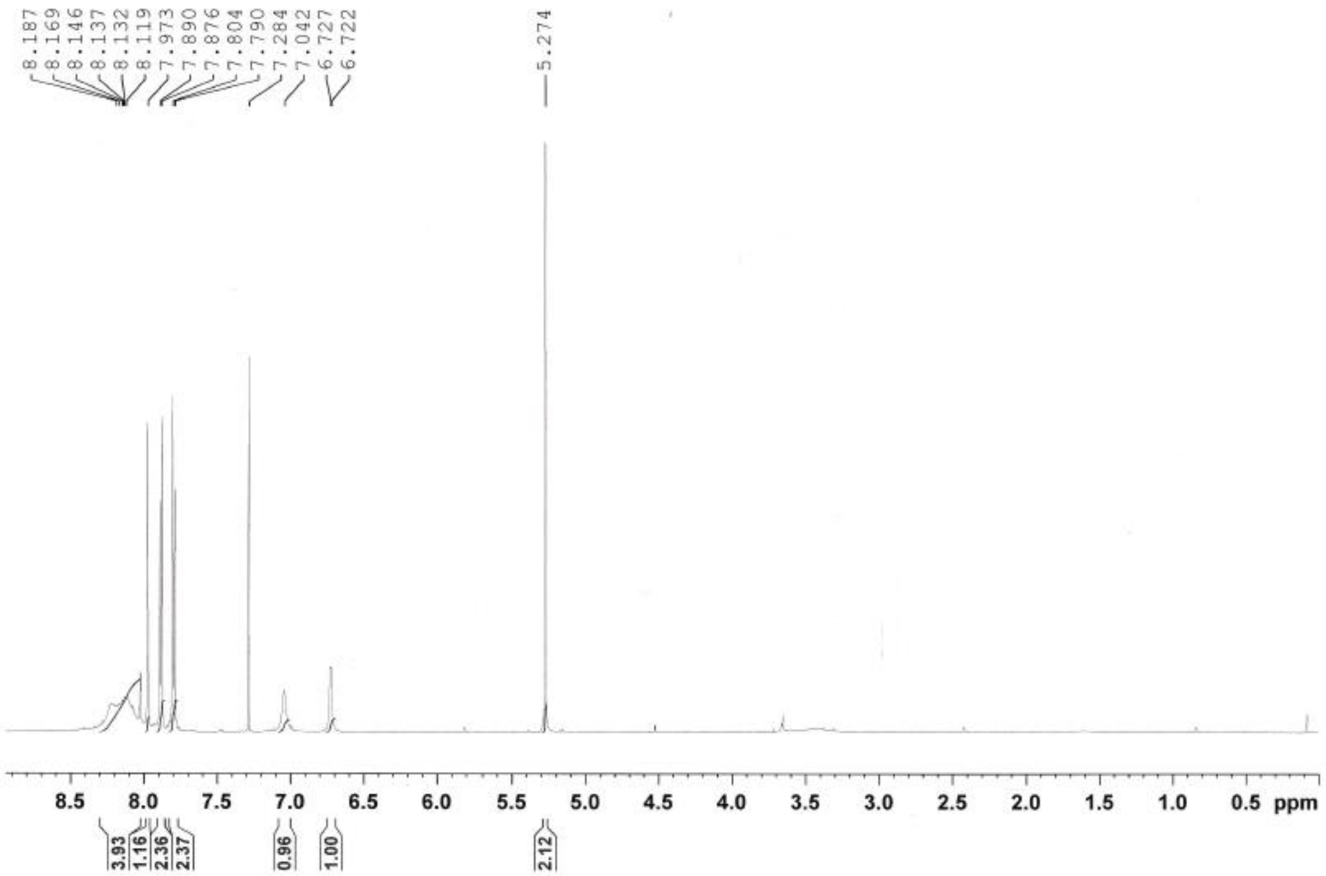

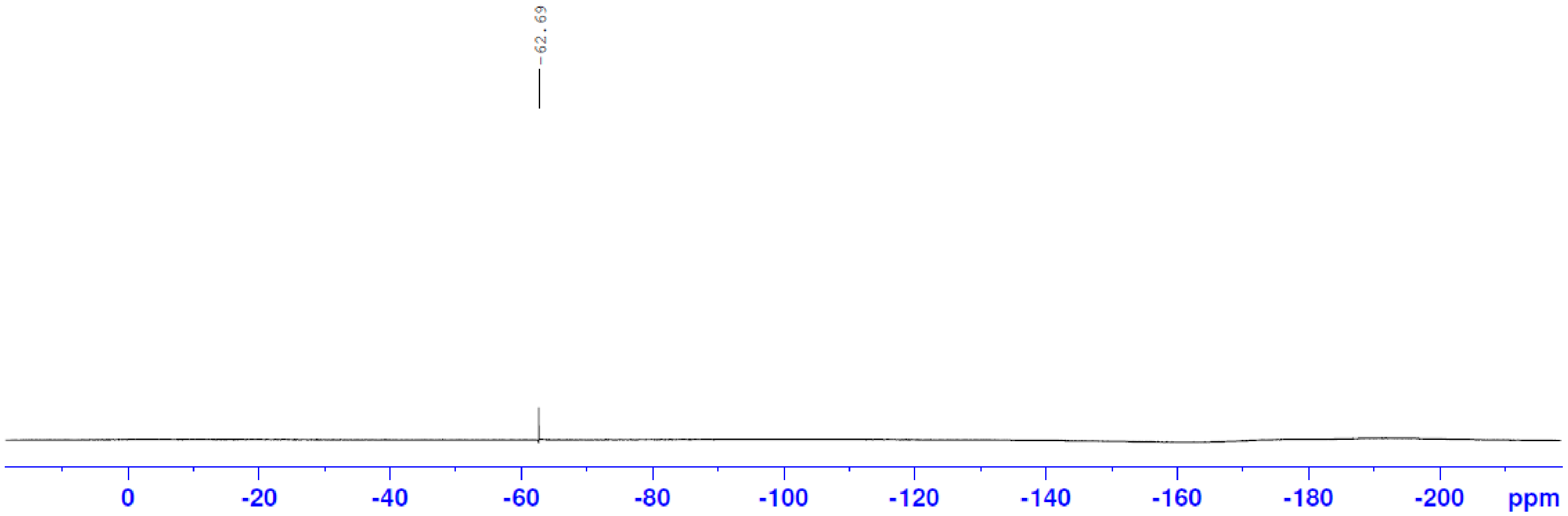
-62.69
0
-20
-40
-60
-80
-100
-120
-140
-160
-180
-200
ppm

Acquisition Parameter

Source Type	ESI	Ion Polarity	Positive	Set Nebulizer	0.3 Bar
Focus	Active	Set Capillary	4000 V	Set Dry Heater	240 °C
Scan Begin	100 m/z	Set End Plate Offset	-500 V	Set Dry Gas	4.0 l/min
Scan End	1000 m/z	Set Charging Voltage	2000 V	Set Divert Valve	Source
		Set Corona	0 nA	Set APCI Heater	0 °C

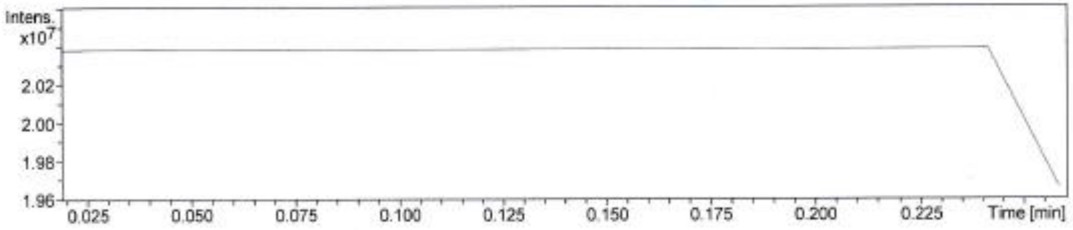

+MS, 0.0-0.2min #2-14

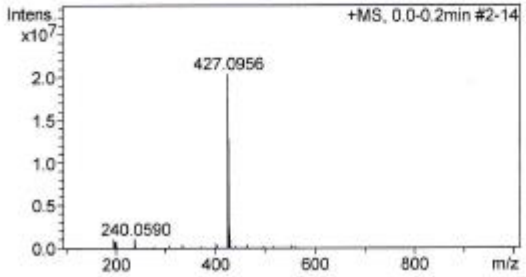

NMR spectra and HR MS of **TDT4b**

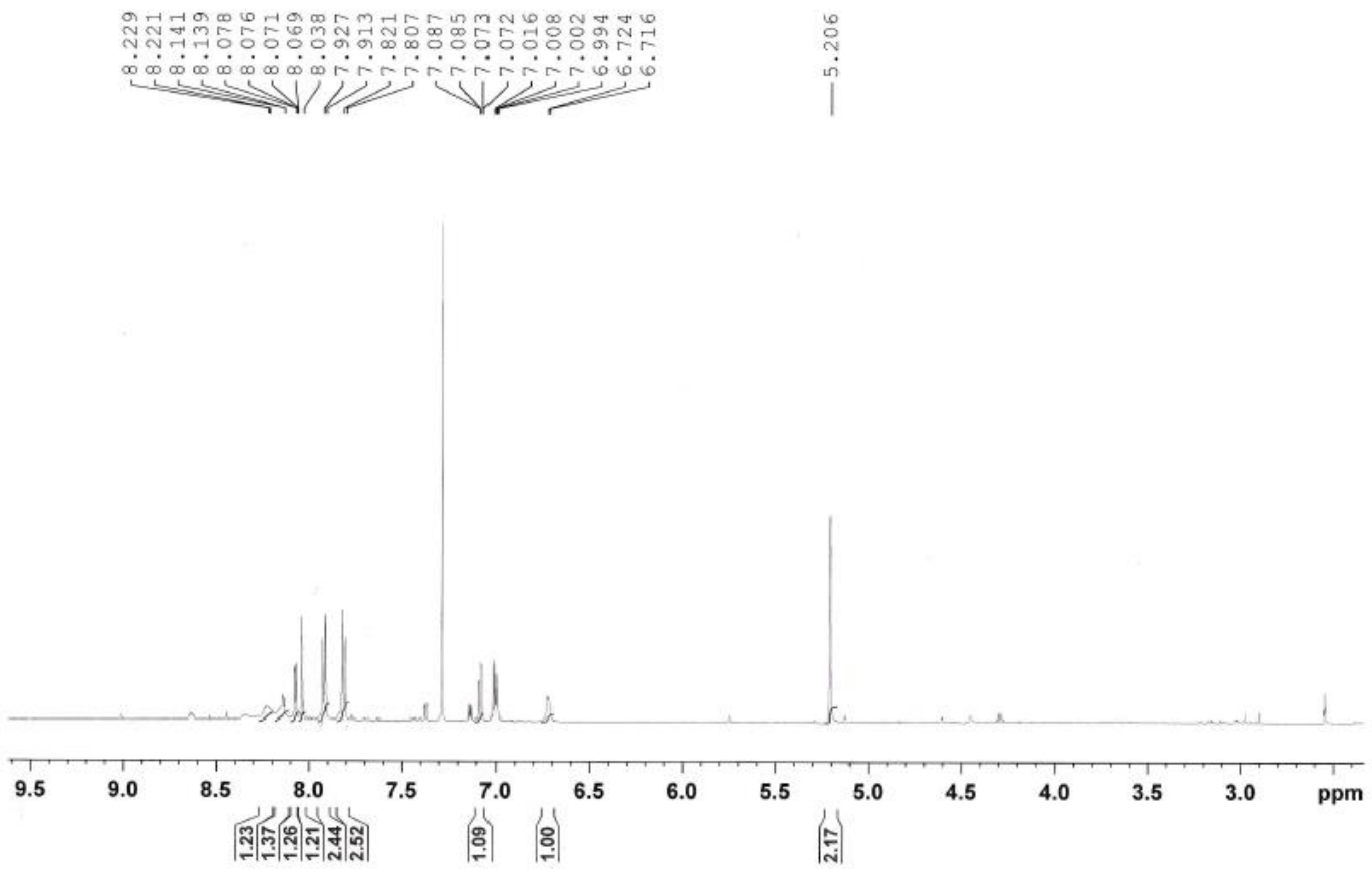

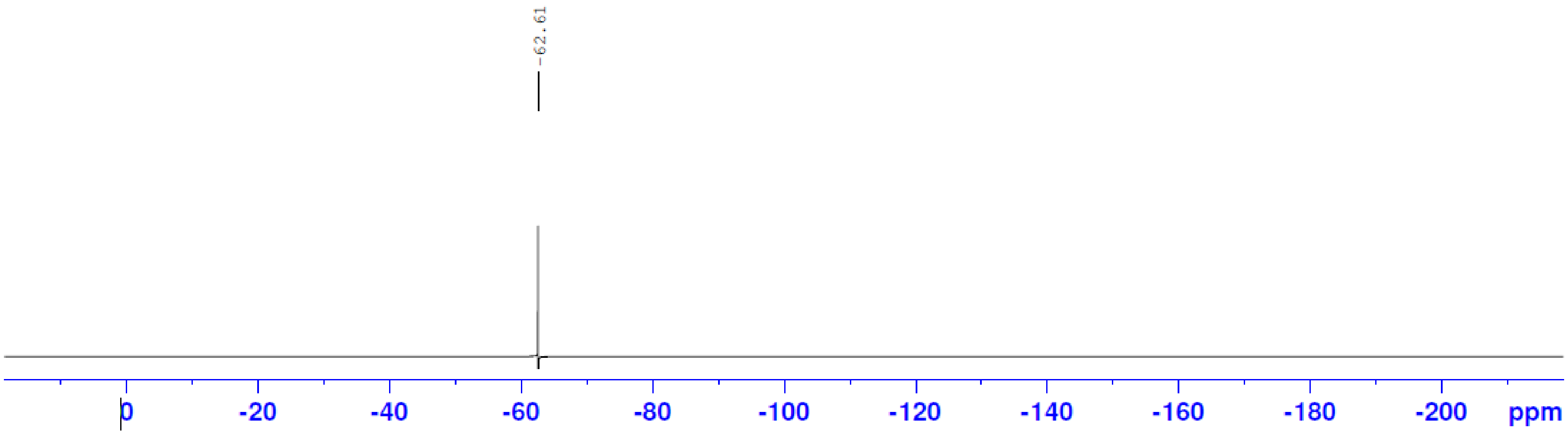
-62.61
0
-20
-40
-60
-80
-100
-120
-140
-160
-180
-200
ppm

Acquisition Parameter

Source Type	ESI	Ion Polarity	Positive	Set Nebulizer	0.3 Bar
Focus	Active	Set Capillary	4000 V	Set Dry Heater	240 °C
Scan Begin	100 m/z	Set End Plate Offset	-500 V	Set Dry Gas	4.0 l/min
Scan End	1000 m/z	Set Charging Voltage	2000 V	Set Divert Valve	Source
		Set Corona	0 nA	Set APCI Heater	0 °C

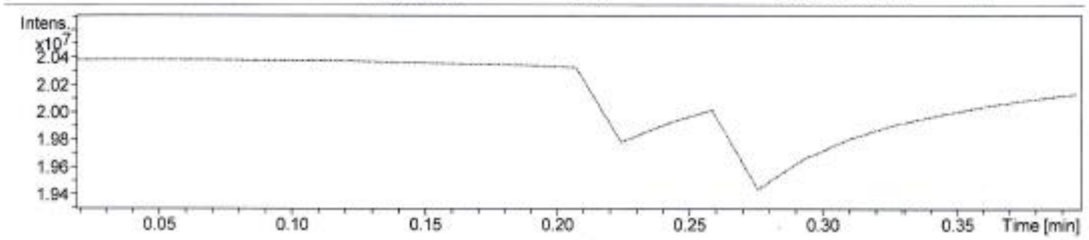

+MS, 0.1-0.4min #4-22

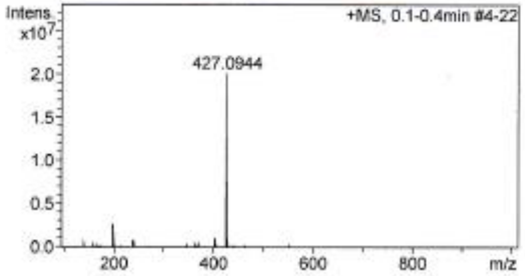

Table S1. RMSD lower bound (LB) and upper bound (UB) results for each docking position.

**TDT2b**			**TDT3b**		
**Docking pose**	**RMSD LB [Å]**	**RMSD UB [Å]**	**Docking pose**	**RMSD LB [Å]**	**RMSD UB [Å]**
1	0.000	0.000	1	0.000	0.000
2	1.480	3.998	2	5.187	7.234
3	4.853	6.031	3	0.801	3.623
4	0.645	3.510	4	5.973	9.640
5	5.729	8.836	5	1.854	4.107
6	5.772	9.371	6	5.410	8.481
7	1.885	2.670	7	1.713	2.310
8	5.275	8.517	8	5.921	9.010
9	5.513	8.766	9	1.788	3.819
10	1.906	3.710	10	5.462	8.891
**MEAN**	3.306	5.541	MEAN	3.411	5.712
**SD**	2.319	3.229	SD	2.371	3.352
